# Solar Trap‐Adsorption Photocathode for Highly Stable 2.4 V Dual‐Ion Solid‐State Iodine Batteries

**DOI:** 10.1002/adma.202504492

**Published:** 2025-08-06

**Authors:** Xueying Zhang, Lingfeng Zhu, Jiale Cao, Zheng Li, Youliang Wang, Jianwei Zhao, Zhencheng Xie, Xiaoning Li, Tianyi Ma, Bo‐Tian Liu

**Affiliations:** ^1^ Guangxi Key Laboratory of Electrochemical and Magneto‐chemical Functional Materials Guilin University of Technology Guilin 541004 China; ^2^ Centre for Atomaterials and Nanomanufacturing (CAN) School of Science RMIT University Melbourne VIC 3000 Australia; ^3^ School of Chemistry and Chemical Engineering Nanchang University Nanchang 330031 China; ^4^ Shenzhen HUASUAN Technology Co., Ltd Shenzhen 518055 China

**Keywords:** four‐electron conversion reaction, iodine‐based batteries, photo‐assisted cathode, shuttle effect, solid‐state battery

## Abstract

Rechargeable aqueous iodine‐based electrochemical energy storage systems offer a cost‐effective alternative to conventional alkali metal batteries for grid‐scale applications. However, their practical deployment is hindered by sluggish iodine redox kinetics and the shuttle of polyiodides, which severely limit their lifespan. To address these challenges, a novel solid‐state organic||I_2_ battery leveraging a Co_3_O_4_‐TiO_2_ heterojunction photocathode is developed. By integrating a photo‐assisted mechanism with an innovative device architecture, the system achieves accelerated iodine conversion kinetics, enhances iodide ion utilization, and enables a four‐electron redox pathway. Theoretical calculation combined with electrochemical analysis reveals that the photo‐assisted mechanism promotes electrostatic adsorption of polyiodides, accelerates interfacial charge transfer, and significantly improves iodine redox kinetics. As a result, the organic||I_2_ battery delivers a high specific capacity of 1.36 mAh cm^−2^, a discharge voltage of 2.4 V, and excellent cycle stability over 1000 cycles, retaining 80.9% of its capacity at a current density of 10 mA cm^−2^. This photo‐enhanced battery exhibits strong competitiveness compared to previously reported iodine‐based batteries. The remarkable performance of this photo‐assisted prototype offers a sustainable and cost‐effective solution for next‐generation energy storage.

## Graphical abstract

A novel organic||I_2_ battery system with a photo‐assisted mechanism is developed. This design offers accelerated iodine conversion kinetics, enhanced utilization of iodine ions, avoided polyiodide's shuttle, and four‐electron reaction mechanism for iodine species conversion. Leveraging the above merits, the photo‐assisted PTCDI||I_2_ battery delivers a high energy density of 0.66 mWh cm^−2^ alongside remarkable long‐term cycling durability and excellent bending capability.

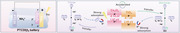

## 1 Introduction

Clean and sustainable solar energy is poised to become an indispensable component of the future global energy landscape.^[^ ^1^, ^2^, ^3^, ^4^, ^5^ ^]^ However, its inherent intermittency and instability pose significant challenges for direct integration with power demand.^[^ ^6^, ^7^, ^8^, ^9^, ^10^, ^11^, ^12^ ^]^ Addressing these issues necessitates flexible solar energy storage systems, but existing options like pumped hydro and thermal tower systems are constrained by geographic and scale limitations. Moreover, the multi‐step conversion process involved in these technologies introduces significant unavoidable energy losses, ultimately reducing the overall conversion efficiency (CE).^[^ ^13^ ^]^ Addressing these challenges calls for the development of electrochemical energy storage systems (ESSs) with higher conversion efficiency to facilitate the effective integration of solar energy into the electrical grid. In this context, effective and affordable ESSs are widely recognized as a key enabler for future grid infrastructure, which is expected to accommodate a high share of renewable energy sources. Among the various types of ESSs, the new rechargeable metal||I_2_ batteries (e.g., Fe||I_2_, Al||I_2,_ and Zn||I_2_) have gained considerable attention due to their cost‐effectiveness, safety, and promising electrochemical performance.^[^ ^14^, ^15^, ^16^, ^17^, ^18^, ^19^ ^]^ Despite their potential, these systems still face critical challenges, including dendrite growth on the metal anode and the shuttling effect of the iodine species during cycling. Recent research efforts have been devoted to modifying the metal anode and immobilizing iodine species within the cathode to mitigate these issues and improve the battery's performance.^[^ ^20^, ^21^, ^22^, ^23^, ^24^, ^25^ ^]^ However, the progress achieved thus far remains limited.

Recently, several novel alternative systems have been proposed, including the replacement of metal anode materials with organic compounds or hydrogen‐based electrodes.^[^ ^26^, ^27^ ^]^ For example, Zhang et al.^[^ ^26^ ^]^ proposed a 3,4,9,10‐perylenetetracarboxylic diimide (PTCDI)||I_2_ battery that exhibited an exceptionally long lifespan (92000 cycles at 40 A g^−1^) and high discharge capacity at high current densities (e.g., 104 mAh g^−1^ at 160 A g^−1^). The organic‐anode strategy effectively suppresses dendrite growth, resulting in superior performance compared to traditional metal||I_2_ batteries. In parallel, there has been an unprecedented interest in integrated photo‐charged electrochemical ESSs.^[^ ^2^, ^28^ ^]^ These systems are designed to convert solar energy directly into electrical energy and store it as chemical energy, thereby minimizing the energy losses inherent in conventional multi‐step conversion processes. Theoretically, the introduction of photo‐generated carriers significantly increases the carrier density within the battery, accelerates charge–discharge kinetics, and promotes more complete redox reactions. Besides, photo‐generated holes can also mitigate the shuttle effect of iodine species, enhancing the overall stability and efficiency of the system. Nevertheless, the application of photo‐assisted strategies in organic||I_2_ battery remains unexplored, representing a promising avenue for future research.

Here, we propose a solid‐state organic||I_2_ battery incorporating a photo‐assisted mechanism. In this system, a dendrite‐free anode is constructed by vacuum filtering PTCDI and carbon nanotubes (CNT) into a composite film (PTCDI/CNT). While a Co_3_O_4_‐TiO_2_ heterojunction is in situ grown on carbon cloth (CC) as the photocathode, and NH_4_Cl‐KI/ (polyvinyl alcohol) PVA gel is applied as electrolyte. The heterojunction features a well‐matched band structure that enables a strong photo‐induced electrostatic adsorption for polyiodides (e.g., I_3_^−^ and I_5_^−^), and an accelerated photoelectricity conversion. Besides, the implementation of four‐electron redox pathway (I^−^/I_2_/ICl) expands the battery's voltage window up to 2.4 V, enabling exceptional electrochemical performance. The results indicate that the photo‐excited holes of Co_3_O_4_‐TiO_2_/CC photocathode can promote polyiodide adsorption, lower the energy barrier for the ICl/I_2_/I^−^ conversion, and accelerate the interfacial charge transfer, thereby significantly improving iodine redox kinetics. This mechanism is well supported by density functional theory (DFT) calculations and electrochemical analysis. Moreover, the stability of solid‐state organic||I_2_ battery is improved by the strong suppression of polyiodides shuttling, attributed to photo‐generated holes in the valence band of Co_3_O_4_‐TiO_2_/CC photocathode. As a result, the solid‐state organic||I_2_ battery delivers a specific capacity of as high as 1.36 mAh cm^−2^ with discharge voltage of 2.4 V, and excellent cycle stability over 1000 cycles, maintaining a high retention of 80.9% at a current density of 10 mA cm^−2^. This work presents a novel and sustainable framework for developing high‐performance iodine‐based energy storage systems.

## 2 Results and Discussion

### 2.1 Morphology and Structure Characterizations of Co_3_O_4_‐TiO_2_/CC Photocathode

**Figure 1:**
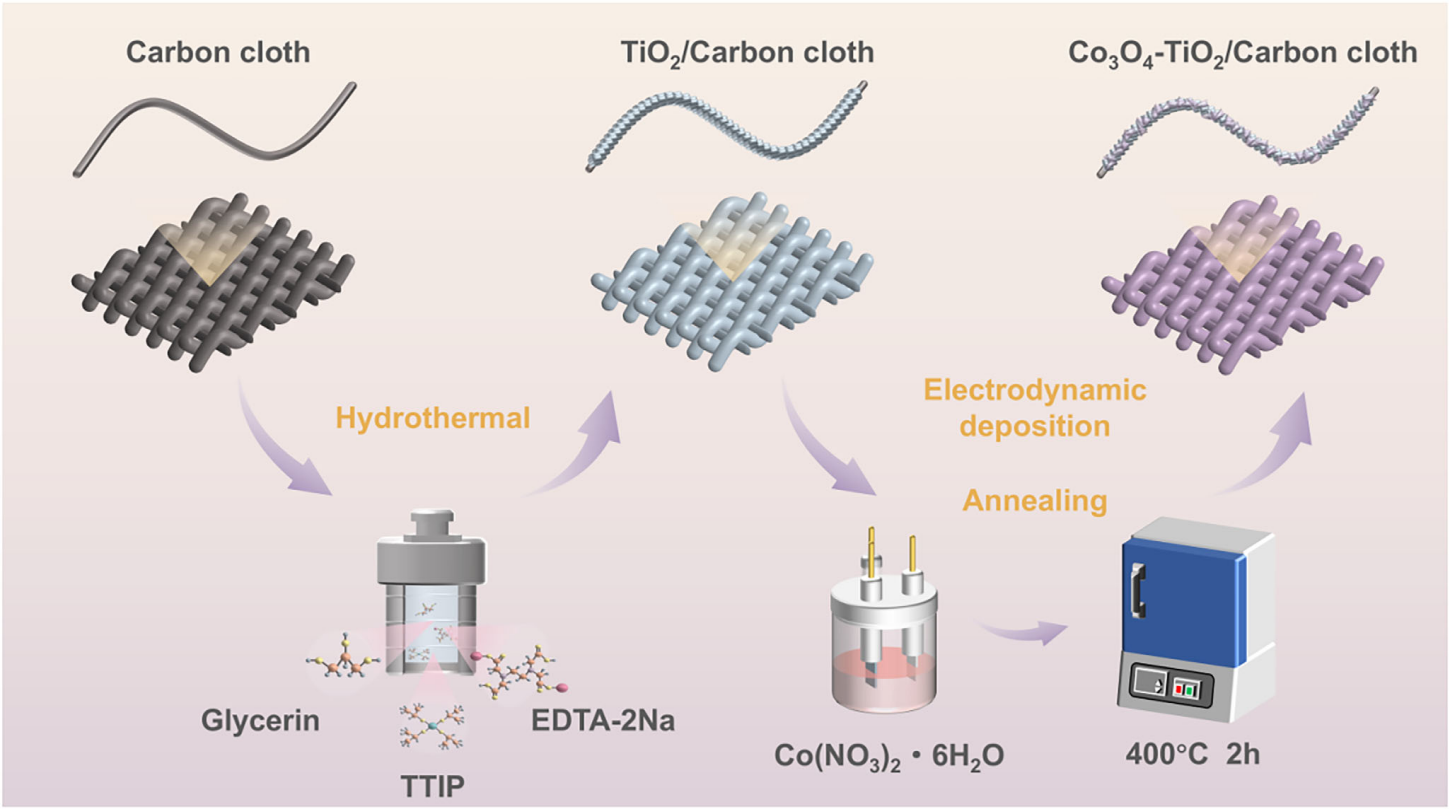
Schematic illustration of the preparation process of Co_3_O_4_‐TiO_2_/CC photocathode.

**Figure 2:**
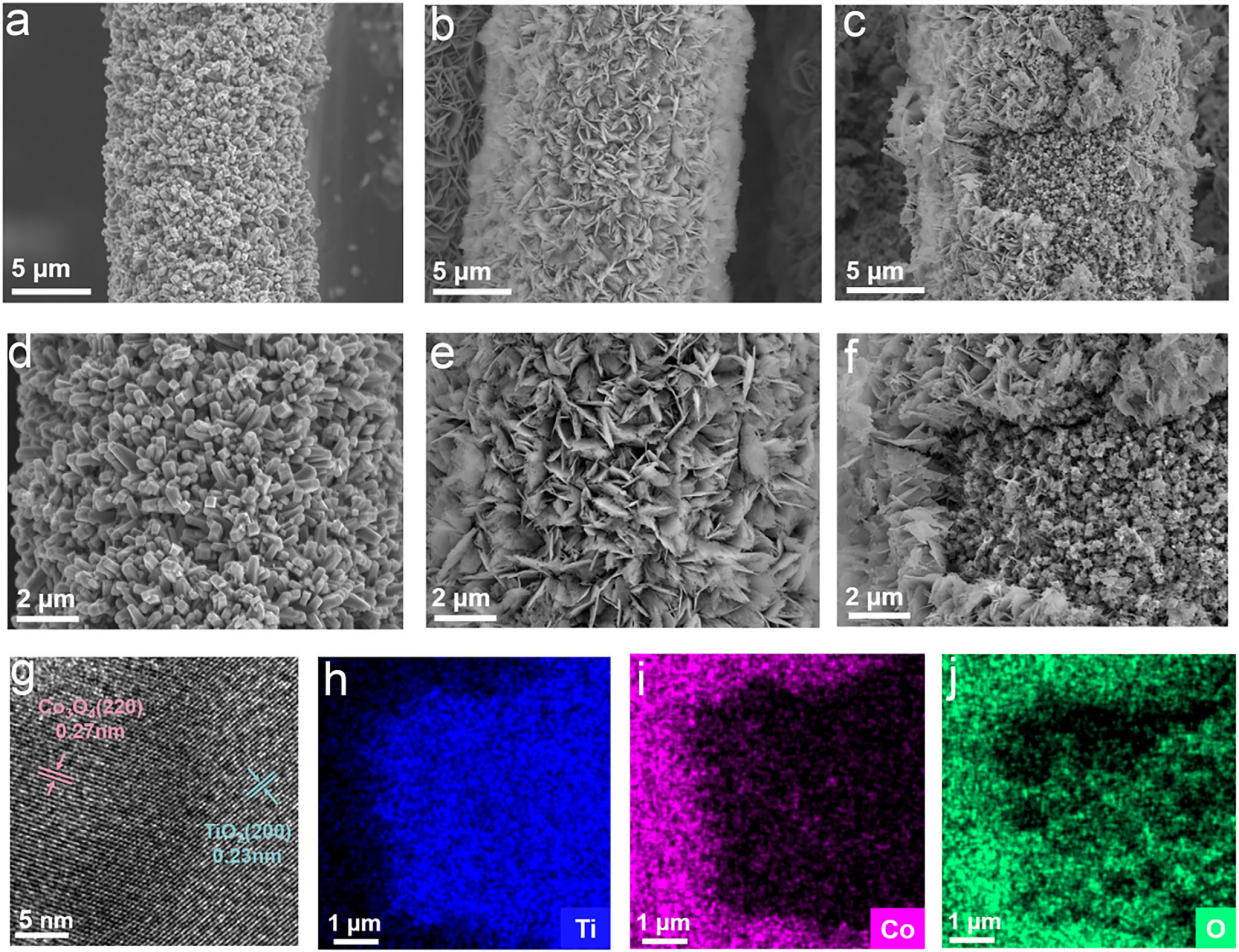
a) Top‐view SEM of TiO_2_/CC; b,c) Top‐view SEM of Co_3_O_4_‐TiO_2_/CC; d) High‐resolution top‐view SEM of TiO_2_/CC; e,f) Top‐view SEM of the Co_3_O_4_‐TiO_2_/CC; g) HR‐TEM of Co_3_O_4_‐TiO_2_/CC heterojunction; h–j) EDS mapping of Co_3_O_4_‐TiO_2_/CC.

As illustrated in **Figure** **1**, the Co_3_O_4_‐TiO_2_/CC photocathode was prepared through a combination of hydrothermal synthesis and electrodynamic deposition. Initially, TiO_2_ nanobar arrays were uniformly grown on CC fibers via a facile hydrothermal process, forming the TiO_2_/CC photocathode. Scanning electron microscopy (SEM) images in **Figure** **2**a,d show that the TiO_2_ nanobar arrays, with an average diameter ranging from 300 to 500 nm, are uniformly distributed across the CC fibers. Subsequently, Co_3_O_4_ was deposited onto the TiO_2_/CC surface via an electrodynamic deposition process, yielding the final Co_3_O_4_‐TiO_2_/CC heterojunction photocathode (Figure 1). As shown in Figure 2b,c, the Co_3_O_4_ layer uniformly coats the surface of TiO_2_ with intimate contact, suggesting the well‐established formation of a heterojunction. This heterojunction is further confirmed by high‐resolution transmission electron microscopy (HR‐TEM), where lattice fringes corresponding to the (220) planes of Co_3_O_4_ and the (200) planes of TiO_2_ are clearly visible, indicating the presence of a semi‐coherent interface (Figure 2g). Moreover, SEM images (Figure 2e,f) demonstrate that the Co_3_O_4_ layer forms a 3D porous structure composed of vertically interlaced nanosheets. This unique architecture is expected to enhance efficient electron and ion transport, thereby improving the kinetics and utilization of iodine ions conversion. The element distribution of Co_3_O_4_‐TiO_2_ photocathodes is further confirmed by energy dispersive spectroscopy (EDS). Elemental mapping images in Figure 2h–j display uniform distributions of Co, Ti and O elements across the photocathode surface.

**Figure 3:**
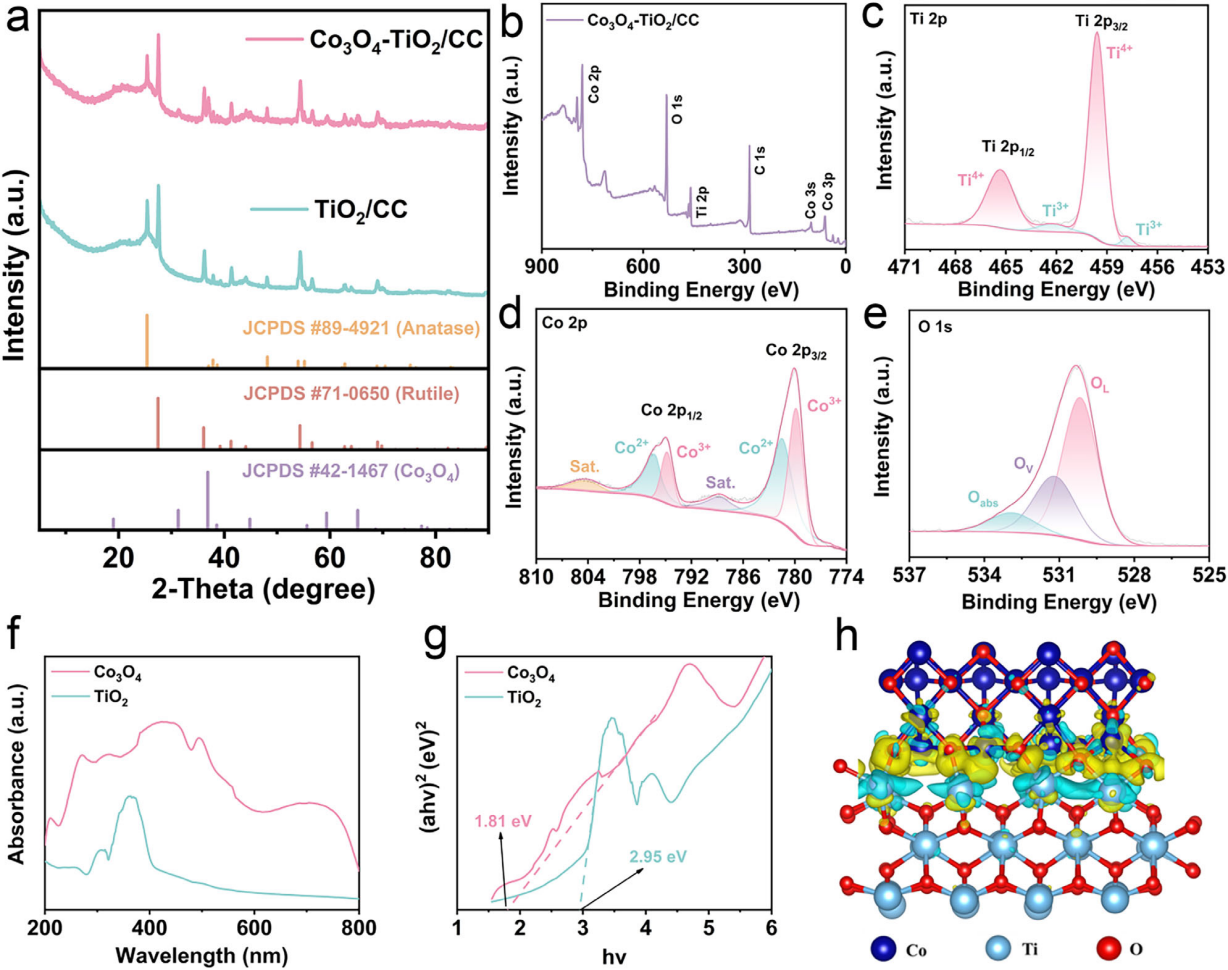
a) XRD patterns of the TiO_2_/CC and the Co_3_O_4_‐TiO_2_/CC; b) XPS spectrum of the Co_3_O_4_‐TiO_2_/CC; c–e) high‐resolution O 1 s, Co 2p and Ti 2p XPS spectra of Co_3_O_4_‐TiO_2_/CC; f) UV–vis spectra of Co_3_O_4_ and TiO_2_; g) Kubelka–Munk energy curve plots of TiO_2_ and Co_3_O_4_; h) Charge density difference for Co_3_O_4_‐TiO_2_ heterojunction based on DFT calculation, with blue representing electron depletion and yellow representing electron gain.

The X‐ray diffraction (XRD) patterns of all samples are presented in **Figure** **3**a, where all diffraction peaks can be well indexed to Co_3_O_4_ (JCPDS Card No. 42–1467) and TiO_2_ phases (JCPDS Card Nos. 71–0650 and 89–4921), with additional peaks corresponding to the CC substrate. These results further confirm the successful structural evolution from the TiO_2_ **/**CC to the Co_3_O_4_‐TiO_2_ **/**CC.^[^ ^29^, ^30^ ^]^ To investigate the surface chemical states, X‐ray photoelectron spectroscopy (XPS) spectra were collected (Figure 3b–e). The peaks of Co, Ti, O elements are clearly shown in the full survey spectrum, which is consistent with the EDS and XRD results. The high‐resolution Ti 2p spectrum is shown in Figure 3c, where two main characteristic peaks at 459.58, 465.18 eV are clearly observed, corresponding to Ti 2p_3/2_, Ti 2p_1/2_ of Ti^4+^ in the TiO_2_, respectively. Two minor peaks at 457.78 and 462.28 eV are attributed to Ti^3+^, likely originating from crystal defects. The high‐resolution Co 2p spectrum displays four peaks at 779.88, 781.58, 794.88 and 796.38 eV, which confirm the coexistence of Co^2+^ and Co^3+^ oxidation states, characteristic of Co_3_O_4_ (Figure 3d).^[^ ^31^, ^32^, ^33^ ^]^ Figure 3e shows three peaks in O 1s spectrum, which are corresponding to absorbed oxygen species (O_abs_), oxygen vacancies (O_V_) and lattice oxygen (O_L_), respectively.^[^ ^34^, ^35^ ^]^ These results collectively confirm the chemical composition and the presence of key functional sites within the Co_3_O_4_‐TiO_2_/CC.

The UV–vis absorption spectra in Figure 3f reveal different light absorption characteristics of Co_3_O_4_ and TiO_2_. The absorption edges of pure Co_3_O_4_ and TiO_2_ are observed at ≈577 and 424 nm, respectively. These values indicate their strong visible light absorption while with different optical responses and distinct band gap energies. Their band gap values (E_g_) are roughly estimated to be 1.81 and 2.95 eV, respectively, based on the equation *ahv* = *A*(*hv*−*Eg*)*^n^* ^/2^, where *n* = 1 for direct bandgap semiconductors (Figure 3g).^[^ ^36^ ^]^ These values confirm that Co_3_O_4_ absorbs more strongly in the visible light region, while TiO_2_ exhibits a wider band gap and absorbs primarily in the UV region. To further investigate their semiconductor properties, the valance band X‐ray photoelectron spectroscopy (VB‐XPS, in Figure S1a, Supporting Information) and Mott–Schottky tests were conducted. As shown in Figure S1b (Supporting Information), the Mott‐Schottky plot of TiO_2_ displays a positive slope, confirming its intrinsic *n*‐type semiconductor behavior, whereas Co_3_O_4_ shows a negative slope, indicating a *p*‐type semiconductor behavior (Figure S1c).^[^ ^37^ ^]^ These findings are consistent with the corresponding VB‐XPS (Figure S1a, Supporting Information) and ultraviolet photoelectron spectroscopy (UPS, Figure S1d,e, Supporting Information). The VB‐XPS analysis (Figure S1a, Supporting Information) shows that the valence band maximum (VBM) of Co_3_O_4_ (0.38 V) lies much closer to the Fermi level compared to that of TiO_2_ (3.56 V), further supporting their respective p‐type and n‐type identities. The UPS measurements further confirm these electronic structures. The secondary electron cutoff and valence band edge positions (Figure S1d,e, Supporting Information) allow for the estimation of work functions and VBM positions, which are used to construct the full band diagrams shown in Figure S1f (Supporting Information). These combined results clearly demonstrate that TiO_2_ is an n‐type semiconductor while Co_3_O_4_ exhibits p‐type behavior.

When Co_3_O_4_ and TiO_2_ come into physical contact to form a heterojunction, electrons transfer from TiO_2_ to Co_3_O_4_, due to the higher Fermi level of TiO_2_ relative to that of Co_3_O_4_. This charge redistribution induces band bending and establishes an internal electric field at the heterointerface, which aligns the energy bands and facilitates subsequent charge carrier dynamics. Upon light irradiation, photogenerated holes in the valence band of TiO_2_ can migrate to Co_3_O_4_, while the corresponding photogenerated electrons remain in the conduction band of TiO_2_. This spatial separation of charge carriers effectively suppresses electron–hole recombination and enhances interfacial charge transfer, thereby accelerating the redox kinetics of polyiodide species involved in the electrochemical reaction. These results further confirm the successful formation of a *p*–*n* junction, which establishes a stable built‐in electric field within the Co_3_O_4_‐TiO_2_ heterojunction. Moreover, based on the combined analysis of Mott‐Schottky plots, VB‐XPS, and UPS spectra, the VBM of Co_3_O_4_ is positioned at ≈1.67 eV (versus normal hydrogen electrode (NHE)), while CBM of TiO_2_ is located around −0.1 eV (vs. NHE). Compared to the I_2_/I^−^ redox potential (≈0.84V versus NHE at PH≈5.6), the CBM of TiO_2_ is sufficiently negative to drive the photoreduction of I_2_ to I^−^, while the VBM of Co_3_O_4_ lies slightly above the I_2_/I^−^ redox potential, enabling hole‐mediated oxidation of I^−^ (Figure S1d–f, Supporting Information). This well‐matched band alignment, between the Co_3_O_4_‐TiO_2_ heterojunction and the redox potential of the iodine species, ensures efficient charge separation, transfer, and regeneration of iodine species during photo‐assisted cycling.

To gain deeper insight into the interfacial interaction and electron transport behavior within the Co_3_O_4_‐TiO_2_ heterojunction, density functional theory (DFT) was performed. The crystal structure of Co_3_O_4_ and TiO_2_ after geometry optimization is shown in Figure S2 (Supporting Information). The charge density difference of the Co_3_O_4_‐TiO_2_ heterojunction in Figure 3h highlights regions of electron accumulation (yellow) and depletion (blue). Notably, significant charge transfer occurs at the interface, particularly between surface Ti atoms in TiO_2_ and O atoms in Co_3_O_4_, indicating remarkable electron transfer from TiO_2_ to Co_3_O_4_. Bader charge analysis quantifies this interaction, revealing that ≈12.76 electrons are transferred from TiO_2_ to Co_3_O_4_. This theoretical result is further verified through XPS depth profiling using an Ar cluster ion beam sputtering system (Figure S3, Supporting Information). Upon initial sputtering, the Co 2p high‐resolution spectrum shows a decrease in the integrated area ratio of Co^3+^/Co^2+^ from 1.11 to 0.57, indicating an increase in electron density in Co_3_O_4_ due to interfacial electron gain. In parallel, the integrated area ratio of Ti^4+^/Ti increases from 0.86 to ≈1.0, consistent with a corresponding electron depletion on TiO_2_. Notably, these altered ratios of Co^3+^/Co^2+^ and Ti^4+^/Ti only revert to values comparable to those of pristine sample after extended sputtering (120s), demonstrating that the observed electron redistribution is primarily localized at the interfacial region. Overall, these results confirm the successful formation of the Co_3_O_4_‐TiO_2_ heterojunction and demonstrate that the built‐in electric field facilitates efficient charge separation and migration, thereby enhancing the redox conversion reactions at the Co_3_O_4_‐TiO_2_ photocathode, as elaborated in the following sections.

The PTCDI/CNT anode was prepared by a sequential process of vacuum filtration and followed by hydrothermal treatment. The PTCDI material was synthesized via the reaction of perylene tetracarboxylic acid dianhydride (PTCDA) with ammonium hydroxide, as detailed in the Experimental Section. The successful conversion of PTCDA to PTCDI is verified by XRD analysis. As shown in Figure S4a (Supporting Information), the main peaks at 11.9°, 24.9°, 27.0°, and 30.3° correspond to the (002), (112), (122) and (132) planes of PTCDI, respectively, consistent with a monoclinic *P21/n* space group.^[^ ^38^, ^39^, ^40^, ^41^, ^42^ ^]^ Further structural characterization was performed using Fourier transform infrared (FTIR) spectroscopy. The main characteristic peak from imide group is located at 1680 cm^−1^, corresponding to the C═O stretching vibration, while other peaks are located at 1590 cm^−1^ (C═C stretching vibration), 1354 cm^−1^ (C─N stretching vibration), 2850 cm^−1^ (C─H stretching vibration), 3040 cm^−1^ (C─H stretching vibration) and 3150 cm^−1^ (N─H stretching vibration), respectively (Figure S4b, Supporting Information). The morphology and structure of PTCDI/CNT anode are further illustrated in Figure S5 (Supporting Information). The PTCDI/CNT anode features a continuous 3D structure composed of PTCDI particles interwoven with long CNTs. This unique micro‐structure endows the PTCDI/CNT anode with remarkable mechanical flexibility, which can be attributed to the synergistic contribution of the flexible CNT framework and the organic PTCDI matrix (Figure S6, Supporting Information).

### 2.2 Reaction Mechanism of Co_3_O_4_‐TiO_2_/CC Photocathode and PTCDI/CNT Anode

**Figure 4:**
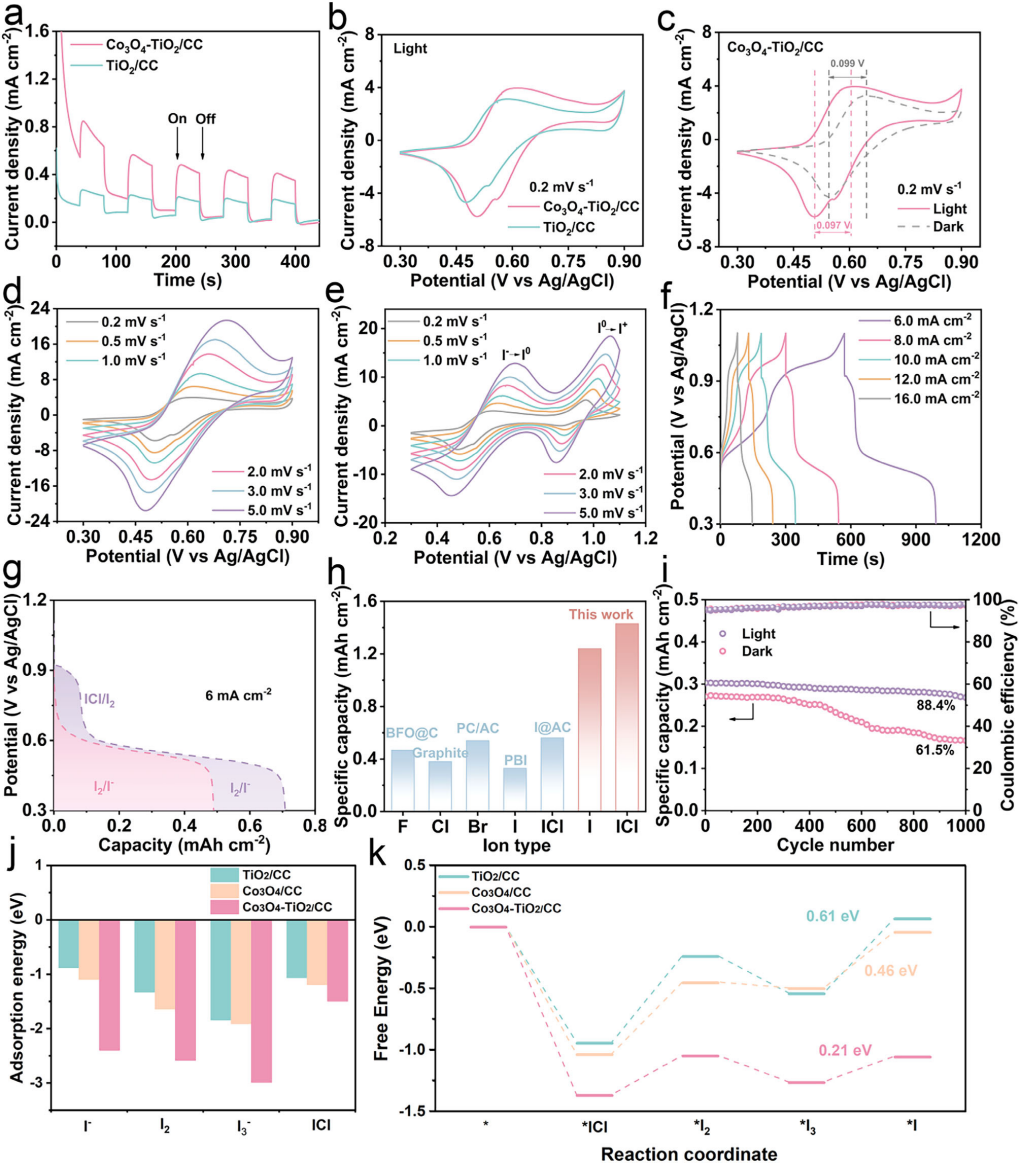
Electrochemical performance of Co_3_O_4_‐TiO_2_/CC photocathode in gel electrolyte. a) Photocurrent spectra of Co_3_O_4_‐TiO_2_/CC photocathode and TiO_2_/CC photocathode; b) CV curves of Co_3_O_4_‐TiO_2_/CC photocathode and TiO_2_/CC photocathode under light at a scan rate of 0.2 mV s^−1^; c) CV curves of Co_3_O_4_‐TiO_2_/CC photocathode under light and in the dark at a scan rate of 0.2 mV s^−1^; d) CV curves of Co_3_O_4_‐TiO_2_/CC photocathode (0.3–0.9 V) under light at various densities range from 0.2 to 5 mV s^−1^; e) CV curves of Co_3_O_4_‐TiO_2_/CC photocathode (0.3–1.1 V) under light at various densities range from 0.2 to 5 mV s^−1^; f) GCD profiles of Co_3_O_4_‐TiO_2_/CC photocathode at various current densities ranging from 6 to 16 mA cm^−2^; g) GCD curves of Co_3_O_4_‐TiO_2_/CC photocathode (0.3–1.1 and 0.3–0.9 V) under light at a current density of 6 mA cm^−2^; h) Comparison of maximum specific capacity of Co_3_O_4_‐TiO_2_/CC photocathode with those halogen‐based cathodes reported in the literature; i) Cycling performances of Co_3_O_4_‐TiO_2_/CC photocathode under light and in the dark at a current density of 10 mA cm^−2^. j) The adsorption energy and k) the Gibbs free energy ladder diagram of different iodine species on Co_3_O_4_‐TiO_2_/CC, TiO_2_/CC and Co_3_O_4_/CC.

To evaluate the electrochemical performance of the Co_3_O_4_‐TiO_2_/CC photo‐assisted cathode and PTCDI/CNT anode, a series of electrochemical measurements, including cyclic voltammetry (CV), galvanostatic charge–discharge (GCD), electrochemical impedance spectroscopy (EIS) and transient photocurrent responses, were conducted in a classical three‐electrode system. The experiments were carried out using NH_4_Cl‐KI/PVA aqueous and gel electrolyte (synthesis detail provided in the Experimental Section), with a saturated Ag/AgCl serving as the reference electrode and a platinum foil serving as the counter electrode. **Figures** **4**a and S7 (Supporting Information) show the transient photocurrent responses of the Co_3_O_4_‐TiO_2_/CC cathode in both electrolyte systems. Notably, the photocurrent observed in the NH_4_Cl‐KI gel electrolyte is significantly higher than that in the aqueous electrolyte, highlighting the superior charge separation efficiency of the Co_3_O_4_‐TiO_2_/CC photocathode when operated in the gel electrolyte. To further evaluate the formation of I_3_^−^, visual observations of color changes in both gel and aqueous electrolytes were recorded at a scan rate of 5 mV s^−1^ (Figure S8, Supporting Information). The aqueous electrolyte exhibits a uniformly distributed brown coloration, typical of I_3_^−^ formation, whereas the gel electrolyte showed no significant color change, implying different diffusion and stabilization behaviors of iodine species in these two electrolyte systems. More importantly, the Co_3_O_4_‐TiO_2_/CC cathode exhibited a higher average coulombic efficiency (≈96.8%) in the gel electrolyte during long‐term cycling than that in aqueous electrolyte (≈82.8%) (Figure 4i; Figure S9, Supporting Information). Comparative performance data for Co_3_O_4_‐TiO_2_/CC and TiO_2_/CC photocathodes in the aqueous electrolyte are provided in Figures S10 and S11 (Supporting Information), further confirming the enhanced charge dynamics afforded by the heterojunction design. Overall, the Co_3_O_4_‐TiO_2_/CC photocathode demonstrates significantly enhanced electrochemical behavior in the gel electrolyte, highlighting the advantages of the gel matrix in facilitating superior electrochemical activity. Besides, within the gel electrolyte, the introduction of Co_3_O_4_ leads to a significant increase in photocurrent, indicating superior photogenerated carrier separation efficiency (Figure 4a).

The excellent electrochemical performance can be largely attributed to the Co_3_O_4_‐TiO_2_/CC photocathode, which generates a large number of photo‐induced holes that effectively adsorb iodine species (e.g., I^−^, and I_3_^−^). Concurrently, the photo‐generated electrons increase the carrier density, thereby enhancing the number of active sites available for the redox reaction of iodine species at the cathode. This dual effect, enhanced adsorption and increased reaction sites, ensures more complete and efficient redox processes, resulting in greatly improved overall electrochemical performance. Meanwhile, the EIS Nyquist plots of photocathodes are utilized to gain further insights into the charge separation mechanisms. As shown in Figure S12 (Supporting Information), the Nyquist plots reveals that the Co_3_O_4_‐TiO_2_/CC photocathode possesses the smallest arc radius, demonstrating the lowest charge transfer resistance. Under light irradiation, the CV curves in Figure 4b and Figure S13 (Supporting Information) reveal that the Co_3_O_4_‐TiO_2_/CC photocathode exhibits a significantly higher discharge capacity of 1.24 mAh cm^−2^, surpassing both TiO_2_/CC (1.03 mAh cm^−2^) and Co_3_O_4_/CC (1.07 mAh cm^−2^). A detailed comparison under different illumination conditions is provided in Table S1 (Supporting Information). This value is substantially higher than the discharge capacity of 0.87 mAh cm^−2^ recorded in the dark (Figure 4c), highlighting the critical role of photo‐assistance. Moreover, the Co_3_O_4_‐TiO_2_/CC photocathode exhibits the lowest polarization voltage (0.097 V), compared to TiO_2_/CC (0.11 V) or Co_3_O_4_/CC (0.114 V) counterparts. Furthermore, the accelerated redox kinetics of iodine species are further revealed by the CV curves. During anodic and cathodic sweeps within the voltage ranges of 0.3–0.9 V versus Ag/AgCl, a pair of well‐defined redox peaks is observed at ≈0.61/0.51 V at a scan rate of 0.2 mV s^−1^, which can be attribute to conversion reaction between I^−^ and I_2_.

Based on above analysis, a two‐electron redox reaction mechanism occurring at the cathode is proposed as follows:

(1) $$\mathrm{Cathode}\mathrm{reaction}:2I^{-}↔I_{2}+2e^{-}$$

As the scan rate increases from 0.2 to 5.0 mV s^−1^, the redox peak voltages of the Co_3_O_4_‐TiO_2_/CC photocathode exhibit minor shifts, indicating a rate‐controlling process for the redox reversibility. Specifically, despite a 25‐fold increase in scan rate, the oxidation and reduction peak voltages shift by only 80 and 60 mV for the Co_3_O_4_‐TiO_2_/CC photocathode, respectively (Figure 4d). Moreover, the Tafel slope of Co_3_O_4_‐TiO_2_/CC cathode under light (152.7 mV dec^−1^) was lower than that in the dark (218.8 mV dec^−1^) in the iodine reduction reaction, which was ascribed to the reduced energy barrier due to photo‐assisted mechanism (Figure S14a, Supporting Information). Based on the Tafel slope of the iodine redox reaction, the activation energy of the iodine redox conversion was calculated by the following equation:^[^ ^43^ ^]^

(2) $$E_{\mathrm{Red}/\mathrm{Ox}}=E_{\mathrm{Red}/\mathrm{Ox}}^{O}\;-\frac{RT}{b}\varphi_{cathode}{\left(Ox/Red\right)}_{IR}$$

where *E*_Red/Ox_ is the activation energy of the oxidation or reduction process, $E_{\mathrm{Red}/\mathrm{Ox}}^{O}$ is the intrinsic activation energy, R is the gas constant, b is the Tafel slope, and φ_*cathode*_ is the irreversible potential during the oxidation or reduction process. In comparison with the dark condition, the energy barriers for the I_2_ reduction and I^−^ oxidation at the Co_3_O_4_‐TiO_2_/CC cathode under light were reduced by 2.07 and 3.03 kJ mol^−1^, respectively (Figure S14b, Supporting Information). These findings provide strong quantitative evidence for the photo‐assisted mechanism and are consistent with the improved redox kinetics observed in the CV measurements.

More importantly, the number of electrons involved in the redox process plays a crucial role in determining the electrochemical performance of the electrode. Figure 4d,e shows the CV curves of the two‐electron (I^−^/I_2_) and four‐electron (I^−^/I_2_/ICl) conversion reactions on the Co_3_O_4_‐TiO_2_/CC photocathode, respectively. The emergence of an additional pair of redox peaks at ≈0.83/1.02 V reveals a further oxidation step from I_2_ to ICl. This behavior is further confirmed by galvanostatic charge–discharge (GCD) curves at different current densities (Figure 4f). To directly compare the impacts of two‐electron and four‐electron conversion reactions on electrode performance, GCD profiles were analyzed at a fixed current of 6 mA cm^−2^ (Figure 4g). Remarkably, the four‐electron conversion reaction of the Co_3_O_4_‐TiO_2_/CC photocathode exhibits a higher specific capacity (0.7 mAh cm^−2^) than the two‐electron conversion reaction (0.5 mAh cm^−2^) at a current density of 6 mA cm^−2^. This finding indicates that the four‐electron conversion of iodine ions offers significant advantages in terms of charge storage capacity.

The I^−^/I_2_/ICl conversion pathway is further supported by the *ex situ* UV–vis absorption spectroscopy. As illustrated in Figure S15 (Supporting Information), during charging to 0.7 V versus Ag/AgCl, a broad absorption band in the range of ≈450 to ≈550 nm appears, corresponding to the formation of I_2_ from I^−^. Upon further charging to 1.1 V, a new absorption peak emerges at ≈335 nm, which is attributed to the formation of ICl interhalogen species, indicating the subsequent conversion of I_2_ to I^+^. Meanwhile, the characteristic absorption band of I_2_ (≈450–550 nm) disappears upon charging to 1.1 V, further supporting the oxidative conversion of I_2_ to I^+.[^ ^26^ ^]^ Upon discharging to 0.3 V, the original spectral features reappear, confirming the high reversibility of the iodine redox process. in situ Raman spectroscopy further validates the I^−^/I_2_/ICl conversion mechanism (Figure S16, Supporting Information). Characteristic peaks corresponding to I‐Cl and I_3_^−^ species gradually intensify during the charging process and diminish upon discharging to 0.3 V, providing additional evidence for the proposed electrochemical pathway. Notably, the absence of detectable Raman signals for molecular I_2_ may be attributed to fluorescence interference from impurities in the aqueous solution under illumination. Moreover, I_2_ likely act as a transient intermediate that rapidly reacts with Cl^−^ to form ICl, preventing its accumulation and direct detection under in situ conditions.

Based on the electrochemical analysis and UV–vis absorption spectroscopy, the four‐electron conversion mechanism at the cathode can be elucidated as follows:

(3) $$\mathrm{Cathode}\;\mathrm{reaction}:2Cl^{-}+2I^{-}↔2\mathrm{ICl}+4e^{-}$$

In addition, the specific capacity of the Co_3_O_4_‐TiO_2_/CC photocathode is benchmarked against recently reported iodine‐based systems^[^ ^44^, ^45^, ^46^, ^47^, ^48^ ^]^ (Table S2, Supporting Information). Notably, our Co_3_O_4_‐TiO_2_/CC photocathode achieves a maximum specific capacity of 1.43 mAh cm^−2^ (0.2 mV s^−1^), significantly surpassing those reported for similar systems (Figure 4h). It also exhibits a significantly higher capacity retention (≈88.4% its initial capacity after 1000 cycles) under continuous light irradiation compared to that in the dark (61.5% its initial capacity after 1000 cycles) (Figure 4i). This impressive performance is primarily attributed to the generation of photo‐generated carriers, which notably increases the carrier density within the entire system, facilitates more complete redox processes, and stabilizes charge–discharge cycling over extended operation.

To gain insight into the interacting mechanisms between Co_3_O_4_‐TiO_2_/CC and the various iodine species involved in the conversion reaction (ICl, I_2_, I_3_^−^, and I^−^), DFT calculations were further performed. As the adsorption capability of the catalyst plays an important role in the conversion process, we calculated the adsorption energy of Co_3_O_4_‐TiO_2_/CC, Co_3_O_4_/CC and TiO_2_/CC for I^−^, I_2_, I_3_^−^, and ICl, respectively, as shown in Figure 4j and Table S3 (Supporting Information). The optimized adsorption configurations are shown in Figures S17–S19 (Supporting Information). Obviously, compared with TiO_2_/CC and Co_3_O_4_/CC photocathodes, Co_3_O_4_‐TiO_2_/CC exhibits the lowest absorption energy toward iodine species, with values of −2.40 eV for I^−^, −2.58 eV for I_2_, −2.99 eV for I_3_^−^ and −1.49 eV for ICl, respectively. This result is consistent with the visual adsorption experiments mentioned above. To further understand the catalytic behaviors of Co_3_O_4_‐TiO_2_/CC in the iodine redox process, the Gibbs free energy (*ΔG*) of the ICl reduction pathways with the reaction intermediates was calculated. As shown in Figure 4k, the *ΔG* for the formation of ^∗^I on Co_3_O_4_‐TiO_2_/CC (−1.05 eV) is significantly more negative than those of Co_3_O_4_/CC (−0.04 eV) and TiO_2_/CC (0.06 eV), indicating a more thermodynamically favorable iodine reduction reaction (IRR) process on Co_3_O_4_‐TiO_2_/CC. Furthermore, for the rate‐determining step of I_3_^−^/I^−^, the Gibbs free energy of Co_3_O_4_‐TiO_2_/CC (0.21 eV) is lower than that of Co_3_O_4_/CC (0.46 eV) and TiO_2_/CC (0.61 eV), indicating that the Co_3_O_4_‐TiO_2_/CC provides the most satisfactory conversion kinetics for iodine species, thereby confirming its highly efficient catalytic activity. In summary, in combined with the aforementioned spectroscopic analyses and theoretical calculation results, the reaction pathway of ICl → I_2_ → I_3_^−^ →I^−^ on Co_3_O_4_‐TiO_2_/CC is well confirmed.

**Figure 5:**
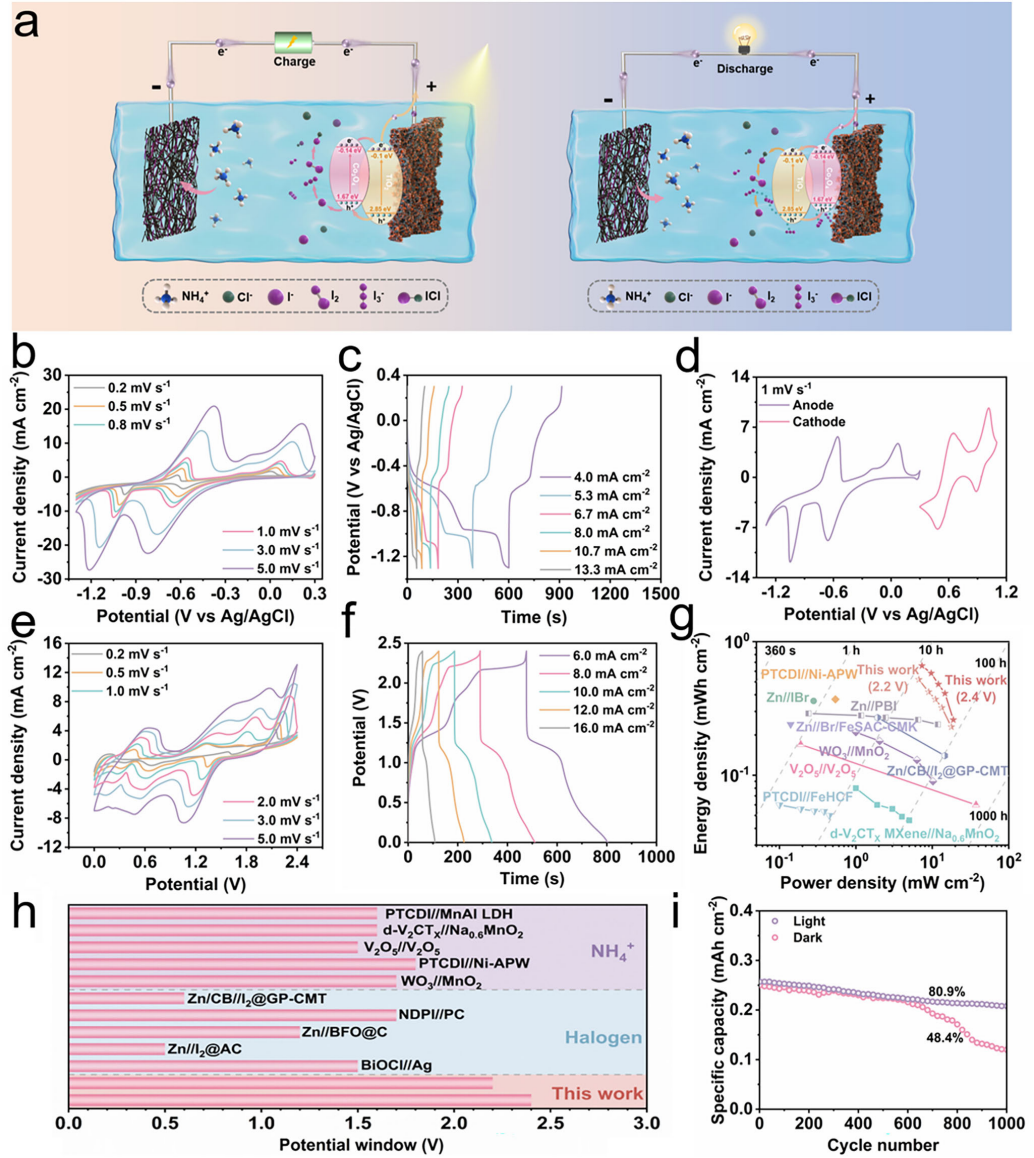
Electrochemical performance of PTCDI/CNT anode and solid‐state PTCDI||I_2_ prototype batteries. a) Schematic illustration of solid‐state PTCDI||I_2_ prototype batteries; b) CV curves of PTCDI/CNT anode at various scan rate from 0.2 to 5 mV s^−1^; c) GCD profiles of PTCDI/CNT anode at various current densities ranging from 4 to 13.3 mA cm^−2^; d) CV curves for PTCDI/CNT anode and Co_3_O_4_‐TiO_2_/CC photocathode in the corresponding positive and negative window at a scan rate of 1 mV s^−1^; e) CV curves of solid‐state PTCDI||I_2_ prototype batteries at various scan rate from 0.2 to 5 mV s^−1^; f) GCD profiles of solid‐state PTCDI||I_2_ prototype batteries at various current densities ranging from 6 to 16 mA cm^−2^; g) Ragone plot of solid‐state PTCDI||I_2_ prototype batteries in comparison to previously reported storage devices; h) Comparison of maximum working windows of solid‐state PTCDI||I_2_ prototype batteries with those halogens and ammonium‐based full cells reported in the literature; i) Cycling performances of solid‐state PTCDI||I_2_ prototype batteries at current density of 10 mA cm^−2^.

In contrast to the high‐capacity Co_3_O_4_‐TiO_2_/CC photocathode, the PTCDI/CNT anode exhibits an ideal battery‐type behavior within a voltage window of −1.3 to 0.3 V versus Ag/AgCl. It achieves a high specific capacitance of 0.57 mAh cm^−2^, which is attributed to a reversible redox reaction involving NH_4_^+^‐induced hydrogen bond. The CV curves of the PTCDI/CNT anode exhibit two pairs of well‐defined redox peaks at −0.38/−1.21 V and 0.21/−0.82 V, which can be attributed to the reversible enolation of the carbonyl groups in the PTCDI structure (**Figure** **5**b). To further elucidate the charge storage mechanism, *ex situ* XRD analysis was carried out on the PTCDI/CNT anode at different states of charge (Figure S20, Supporting Information). The observed reversible shift in (020) diffraction peak indicates periodic lattice expansion and contraction during the charge/discharge process, which is consistent with the insertion/extraction of NH_4_^+^.^[^ ^42^ ^]^ This structural evolution aligns well with the redox processes observed in the CV curves, providing direct evidence for the ion intercalation mechanism governing the electrochemical reaction. As shown in Figure S21 (Supporting Information), this redox process involves the reversible incorporation of two NH_4_^+^ ions, further showing the critical role of NH_4_^+^ in the charge storage mechanism. Moreover, the distinct separation between the two pairs of redox peaks suggests varying degrees of polarization during the electrochemical process. This behavior is likely due to the competitive interaction between NH_4_^+^ and H^+^ ions at the electrode‐electrolyte interface. Supporting this interpretation, a small peak appears between the two major oxidation peaks, suggesting the involvement of H^+^ in the redox reaction. While such features are commonly observed in studies of PTCDI‐based anodes, their exact origin remains not fully understood, likely due to the complex bonding and electronic structures inherent to organic materials. We hypothesize that this small peak is associated with side reactions involving H⁺ ions, especially given the mildly acidic nature of the electrolyte (pH ≈5.6). Specifically, H^+^ ions may interfere with the reversible NH_4_^+^ intercalation‐deintercalation process. H^+^ ions can have a “pseudo‐promoting” effect on the deintercalation of NH_4_^+^ from the electrode material.^[^ ^49^, ^50^ ^]^ Owing to their smaller ionic radius, H^+^ ions may also be co‐intercalated along with the reaction during NH_4_^+^ intercalation. However, when cations are extracted, some adsorbed H^+^ ions surface may be preferentially extracted from the PTCDI surface instead of NH_4_^+^, resulting in incomplete deintercalation and a reduced specific charge capacity. This competitive mechanism contributes to the relatively low Coulombic efficiency and the differences observed in the redox plateaus.

Moreover, the redox peaks positions in the CV curves correlate well with the voltage plateaus in the GCD profiles at various current densities (Figure 5c). EIS measurements further support above‐mentioned results (Figure S22a, Supporting Information). The interfacial resistance (*R*_i_, 1.5 Ω) and charge‐transfer resistance (*R*_ct_, 10.4 Ω) are very small, which is consistent with IR drop results observed. Such outstanding electrochemical properties are attributed to the self‐standing electrodes with the integrated interfacial structure, which facilitates electron and ion transport within the electrode. Notably, the presence of both NH_4_^+^ and K^+^ ions in electrolytes raises the possibility of competition during charge/discharge process. To further reveal the specific cation species stored, GCD measurements were performed on PTCDI/CNT at various current densities (Figure S22b, Supporting Information). The results confirm that the PTCDI primarily reacts with NH_4_^+^ rather than K^+^, consisting with these previous studies.^[^ ^26^, ^42^, ^51^, ^52^, ^53^, ^54^, ^55^ ^]^ According to the aforementioned results, the electrochemical reactions of PTCDI anode are proposed as follows:

(4) $$Anode\;reaction:4PTCDI+4N{H_{4}}^{+}+4e^{-}↔4PTCDI-NH_{4}$$

### 2.3 Demonstration of a 2.4 V PTCDI||I_2_ Prototype Battery

A solid‐state PTCDI||I_2_ prototype battery was constructed using classical redox pair at both cathode (I^−^+4e^−^↔ICl^−^) and anode (4PTCDI+4NH_4_^+^+4e^−^↔4PTCDI‐NH_4_), all operating within a gel electrolyte. It should be noted that this PTCDI||I_2_ device employs a dual‐ion energy storage mechanism, wherein cations are reversibly stored in the anode and anions are stored in the cathode. This dual‐ion configuration offers a promising avenue for boosting the overall energy density and operational stability of the battery. Detailed information on the cell configuration and working mechanisms of solid‐state PTCDI||I_2_ batteries is provided in Figure 5a, which offers insights into the synergistic interactions that enhance battery performance. The overall electrochemical reaction of PTCDI||I_2_ prototype battery can be expressed as:

(5) $$\begin{array}{ccc} Battery\;reaction: & & 4PTCDI+4N{H_{4}}^{+}+2Cl^{-}+2I^{-}\\ & & ↔4PTCDI-NH_{4}+2ICl \end{array}$$

To ensure optimal charge balance between the two electrodes, the mass ratio of PTCDI in the anode was precisely adjusted. As demonstrated in Figure 5d, comparative CV curves of the anode and cathode show well‐matched charge capacities, indicating effective charge balancing. Leveraging these advantages, the working windows of solid‐state PTCDI||I_2_ batteries is increased up to 2.4 V, which is higher than that of previously reported halogens and ammonium‐based full cells (Figure 5h).^[^ ^18^, ^42^, ^44^, ^46^, ^48^, ^56^, ^57^, ^58^, ^59^, ^60^ ^]^ It is noteworthy that the solid‐state PTCDI||I_2_ device exhibits several well‐defined redox pairs in its CV profiles, which is consistent with GCD profiles at various current densities (Figure 5e,f). Although a slight discrepancy between charge and discharge capacities is observed at low current densities, the system demonstrates significantly improved reversibility at higher rates. This behavior can be primarily attributed to diffusion‐limited kinetics during ion insertion and extraction.^[^ ^52^ ^]^ Besides, the battery achieves a maximum specific capacity of 1.36 mAh cm^−2^ at a scan rate of 0.2 mV s^−1^. Combined with the merits of high voltage window and high specific capacity, the solid‐state PTCDI||I_2_ battery achieve a maximum energy density of 0.66 mWh cm^−2^, surpassing that of reported ammonium and iodine‐based batteries (calculating by the areal of cathode) (Figure 5g; Table S4, Supporting Information). This detailed comparison includes Zn/CB//I_2_@GP‐CMT (0.27 mWh cm^−2^ at 1.93 mW cm^−2^),^[^ ^18^ ^]^ PTCDI//Ni‐APW (0.37 mWh cm^−2^ at 0.54 mW cm^−2^),^[^ ^42^ ^]^ Zn//PBI (0.29 mWh cm^−2^ at 0.24 mW cm^−2^),^[^ ^47^ ^]^ d‐V_2_CT*_x_* MXene//Na_0.6_MnO_2_ (0.08 mWh cm^−2^ at 1 mW cm^−2^),^[^ ^56^ ^]^ Bi‐layered V_2_O_5_//Bi‐layered V_2_O_5_ (0.17 mWh cm^−2^ at 0.19 mW cm^−2^),^[^ ^58^ ^]^ WO_3_//MnO_2_ (0.21 mWh cm^−2^ at 1 mW cm^−2^),^[^ ^60^ ^]^ PTCDI//FeHCF (0.06 mWh cm^−2^ at 0.1 mW cm^−2^),^[^ ^61^ ^]^ Zn//IBr (0.36 mWh cm^−2^ at 0.28 mW cm^−2^)^[^ ^62^ ^]^ and Zn//Br/FeSAC‐CMK (0.24 mWh cm^−2^ at 0.14 mW cm^−2^).^[^ ^63^ ^]^

To further demonstrate the advantages of solid‐state PTCDI||I_2_ battery, its cycling stability was further investigated by GCD profiles at current density of 10 mA cm^−2^, as shown in Figure 5i. The devices exhibit a significantly higher capacity retention of ≈80.9% of their initial capacity after 1000 cycles under illumination, in sharp contrast to the 48.4% capacity retention observed in darkness (refer to Table S5 and Figure S23a, Supporting Information for detailed comparisons). The solid‐state PTCDI||I_2_ battery also exhibits excellent charge retention, with a minimal voltage loss of less than 0.0033 V h^−1^, indicating negligible self‐discharge (Figure S23b, Supporting Information). Furthermore, the photocurrent response spectra of the Co_3_O_4_‐TiO_2_/CC photocathode after 1000 charge–discharge cycles show only negligible attenuation compared to the initial performance, confirming the long‐term efficiency of photo‐induced charge separation (Figure S24a, Supporting Information). XRD patterns before and after cycling exhibit no notable structural changes, suggesting excellent structural integrity and strong resistance to photodegradation (Figure S24b, Supporting Information). These results confirm that the heterojunction photocathode maintains both photochemical activity and crystallographic stability under long‐term illumination and electrochemical operation.

To further evaluate the long‐term stability of gel electrolyte and the influence of temperature on ion diffusion properties, both capacity retention and EIS spectra in the low‐frequency Warburg region were compared before and after 1000 charge–discharge cycles at 60 °C (Figure S25a,b, Supporting Information). The results demonstrate that the gel electrolyte retains good ion transport characteristics after 1000 cycles, with only minimal changes observed in the low‐frequency Warburg region, indicating stable interfacial ion diffusion under operational conditions. Impressively, the capacity retention of 84.5% at 60 °C is nearly comparable to that at room temperature, suggesting negligible thermal degradation of the gel electrolyte. This observation is further supported by thermogravimetric‐differential scanning calorimetry (TG‐DSC), which reveals no significant weight loss within the typical operational range of 30–70 °C (Figure S25c, Supporting Information). Moreover, negligible dehydration was observed after seven days of static storage at 60 °C, further confirming the structural integrity of the gel electrolyte (Figure S25d, Supporting Information). These results provide compelling evidence that the photo‐assisted mechanism not only facilitates efficient conversion of polyiodides but also effectively prevents the shuttle effect in full batteries.

In parallel, the photoelectric conversion efficiency of the device was calculated to be 3.21%, based on the ratio of photo‐enhanced capacity to the incident light energy. A detailed comparison in Table S6 (Supporting Information) demonstrates that our system achieves a competitive and promising level of photoconversion efficiency under visible light. It is worth noting that the solid‐state devices offer exceptional mechanical flexibility, enduring bending and twisting from 0° to ≈180° without observable structural damage or performance degradation, indicating their suitability for a wider range of environments (Figure S26, Supporting Information). From a material perspective, PTCDI, used as the primary electrode component, is an organic compound with significant advantages in terms of raw material availability, low cost, and synthetic scalability. Although cobalt‐based materials are known for their resource limitations, our design employs an ultra‐low cobalt mass loading (≈1 mg cm^−2^, ≈0.000027 USD cm^−2^), which raises no significant concerns regarding cost or environmental impact, thereby supporting its practical applications. We also notice that cobalt is a highly recyclable strategic metal, with hydrometallurgical methods with 80%–95% recovery efficiency from spent batteries. Overall, these findings underscore the great potential of the solid‐state PTCDI||I_2_ battery system for practical application.

## 3 Conclusion

In summary, we have developed an organic||I_2_ battery system that incorporates a photo‐assisted mechanism. The synergistic advantages of device design offer multiple benefits, including accelerated iodine conversion kinetics, enhanced utilization of iodine ions, and a four‐electron reaction pathway for iodine species conversion. Leveraging these advantages, the photo‐assisted PTCDI||I_2_ battery achieves a high energy density of 0.66 mWh cm^−2^, remarkable long‐term cycling durability, and excellent mechanical flexibility, enduring 180° bending and twisting without damage. This work not only showcases the broad potential of photo‐assisted strategy but also provides valuable insights into the real‐world applications of economical iodine‐based batteries that are safe, eco‐friendly, and economically viable.

## 4 Experimental Section

### Materials

Glycerinum (C_3_H_8_O_3_), 3,4,9,10‐perylenetetracarboxylic dianhydride (PTCDA), ammonia (NH_3_·H_2_O), ethanol (CH_3_CH_2_OH), disodium ethylenediaminetetraacetate (EDTA‐2Na), titanium (IV) isopropoxide (TTIP), cobalt nitrate hexahydrate (Co(NO_3_)_2_·6H_2_O), carbon nanotubes (diameter of 10–20 nm, CNT) and sodium dodecylbenzenesulfonate (C_12_H_25_C_6_H_4_SO_3_Na, SDS) were purchased from Sigma‐Aldrich Chemical Reagent Co., Ltd., all of which were of analytical grade. Distilled water was used for all the aqueous solutions involved in the experiment and during the washing process.

### Preparation of TiO_2_/CC Photocathode and Co_3_O_4_‐TiO_2_/CC Photocathode

The TiO_2_ **/**CC photocathode was fabricated through a hydrothermal technique. Briefly, 0.45 mL TTIP was added to 2 mL glycerinum under vigorous stirring for 30 min to form solution A. Then, 0.89 g EDTA‐2Na was dissolved in 38 mL H_2_O and stirred for 30 min until a transparent solution was obtained (noted as Solution B). In the following step, the as‐prepared solution A and solution B were stirred for 30 min to form the reaction solution. Both the carbon cloth (CC, CeTech, thickness: ≈0.33 mm) and reaction solution were transferred into 50 mL Teflon‐lined stainless‐steel autoclave and kept at 200 °C for 8 h. After cooling to room temperature, the samples were taken out and washed several times with distilled water and ethanol. Finally, the samples were dried in a vacuum oven at 80 °C for 12 h and cut into small pieces with a size of 1 × 1 cm^−2^ as photocathodes. The loading of TiO_2_ on carbon cloth was estimated to be ≈3.5 mg cm^−2^.

In the following electrodynamic deposition process, the Co_3_O_4_‐TiO_2_ heterojunction was constructed on carbon cloth as Co_3_O_4_‐TiO_2_/CC photocathode. Typically, the Co_3_O_4_ was electrodeposited on as‐prepared TiO_2_ electrode using a classic three‐electrode setup at −1.0 V for 10 min in a solution containing 5 mmol Co(NO_3_)_2_ and 30 mL deionized water. A platinum foil and an Ag/AgCl electrode were used as the counter and reference electrode, respectively. Then, the samples were washed several times with water and ethanol and dried at room temperature in air. Finally, the samples were heated to 400 °C in a Muffle furnace and annealed for 2 h. The loading of Co_3_O_4_‐TiO_2_ on carbon cloth was estimated as ≈4.5 mg cm^−2^.

### Preparation of PTCDI/CNT Anode

The PTCDI/CNT anode was prepared by combining the methods of vacuum filtration and hydrothermal reaction. First, 0.2 g PTCDA was dissolved in 12 mL H_2_O under vigorous stirring for 30 min. Then, 3 mL NH_3_·H_2_O was added to the above solution, which was transferred into a 50 mL Teflon‐lined stainless‐steel autoclave and kept at 170 °C for 19 h. After cooling to room temperature, the PTCDI powders were collected by filtration and washed several times using distilled water and ethanol. The collected PTCDI powders were then dried under vacuum at 60 °C for 8 h. Subsequently, 0.05 g PTCDI powders and 0.05 g CNT were mixed by hand grinding in a mortar for 30 min. The 0.1 g SDS and the obtained mixture were then added to 100 mL H_2_O to form a homogeneous slurry. Finally, the PTCDI/CNT anode was obtained using ultrasonication and vacuum filtration, followed by drying at 60 °C for 5 h and cut into small pieces of size 1 × 1 cm^−2^. The loading of PTCDI was estimated as ≈4 mg cm^−2^.

### Fabrication of PTCDI||I_2_ Prototype Batteries

The aqueous PTCDI||I_2_ batteries were constructed with the PTCDI/CNT anode and Co_3_O_4_‐TiO_2_/CC photo‐cathode in opposition to each other in 1 m NH_4_Cl+0.1 m KI aqueous electrolyte. For the solid‐state configuration, a NH_4_Cl–KI/PVA gel electrolyte was prepared by dissolving 4 g of PVA (type 1788), 2.14 g of NH_4_Cl, and 0.66 g of KI in 40 mL of deionized water. The solution was stirred vigorously at 85 °C for 1 h until a homogeneous and transparent sol was obtained. After cooling to room temperature, this gel was cast directly onto the electrode surface and used as both electrolyte and separator in the solid‐state battery assembly. The PTCDI/CNT anode and Co_3_O_4_‐TiO_2_/CC photocathode were then assembled face‐to‐face and allowed to solidify to complete the solid‐state battery.

### Materials Characterization

The morphologies and microstructures of products were characterized by a field‐emission transmission electron microscope (FE‐TEM, JEOL JEM‐2100F, 200 keV) equipped with an energy dispersive X‐ray spectrometer (EDS) and a scanning electron microscope (SEM, Zeiss, 5.020.0 kV). The crystallography and chemical composition of the products were investigated by a powder X‐ray diffraction (Bruker D8 Advance diffractometer with a Cu‐Kα radiation source) and Fourier‐transform infrared (FT‐IR) spectroscopy in the range of 4000–500 cm^−1^ (NICOLET 5397, AVATAR 360 FTIR spectrometer). X‐ray photoelectron spectroscopy (XPS) and VB‐XPS were carried out on the Thermo Scientific K‐Alph system. XPS depth profiling was performed by using a continuous Ar cluster ion beam sputterer. UV–vis–NIR spectroscopy was recorded using a Shimadzu UV‐3600 spectrophotometer at room temperature. in situ Raman spectrum was collected using Thermo Nicolet Almega XR Dispersive Raman Spectrometer, with 785 nm laser. TG‐DSC analysis was performed using Netzsch STA 449 with air flow at a heating rate of 10 °C min^−1^ from room temperature to 200 °C.

### Electrochemical Measurements

The electrochemical performance of the as‐prepared electrodes was conducted using a classic three‐electrode configuration, with platinum foil as the counter electrode, Ag/AgCl electrode as the reference electrode, and NH_4_Cl‐KI as aqueous electrolyte or NH_4_Cl‐KI/PVA as gel electrolyte (1 M NH_4_Cl+ 0.1 M KI). The PTCDI||I_2_ were assembled with Co_3_O_4_‐TiO_2_/CC as cathode and PTCDI/CNT as anode, using NH_4_Cl‐KI aqueous or NH_4_Cl‐KI/PVA gel as electrolyte. This eliminates the need for a separator. The cyclic voltammetry (CV) curves, galvanostatic charge–discharge (GCD) profiles, and electrochemical impedance spectroscopy (EIS) spectra were conducted on an Iviumstat electrochemical analyzer at room temperature. The CV curves and GCD profiles were performed in potential windows from 0.3 to 0.9 V, 0.3 to 1.1 V, −1.3 to 0.3 V, and from 0 to 2.4 V at various scan rates and current densities, respectively. Self‐discharge behavior was evaluated by charging the solid‐state PTCDI||I_2_ battery to 2.4 V and monitoring the open‐circuit potential over 60 h. The photocatalytic experiments were conducted using a 500 W xenon light source with an irradiation wavelength range of 400–800 nm. The distance between the light source and the solution is ≈15 cm.

The specific capacitance (C_S_), energy density (E), and power density (P) from the charge/discharge curves can be calculated in terms of Eqs. (6)–(10)

(6) $$C_{electrode}\left(F.g^{-1}\right)=\frac{Q}{\;A*2V}=\frac{1}{2AvV}\;\int_{V-}^{V+}i\left(V\right)dV$$

(7) $$C_{electrode}\;\left(\mathrm{mAh}.g^{-1}\right)=\frac{V*\;C_{electrode}\left(F.g^{-1}\right)}{3.6}$$

(8) $$C_{electrode}\;\left(\mathrm{mAh}.g^{-1}\right)=\frac{I*\;t}{3600*A}$$

(9) $$E_{device}=\frac{V*\int_{V-}^{V+}i\left(V\right)dV}{\;A*3600*v}$$

(10) $$P_{device}=\frac{\;E}{t}\;*3600$$

where Q is the total voltammetric charge obtained by integrating the positive and negative sweeps (*i*(V) is the current) of a CV curve, *v* is the scan rate, and V represents the potential window (V = V^+^‐V^−^); t is the discharge time; and A is the area of anode or cathode. The energy/power density of the devices is based on the area of cathode.

*DFT Calculation*: All of the calculations were performed in the framework of the spin‐polarized density functional theory with the projector augmented plane‐wave method, as implemented in the Vienna ab initio simulation package (VASP).^[^ ^64^, ^65^ ^]^ The generalized gradient approximation (GGA) proposed by Perdew, Burke, and Ernzerhof (PBE) was selected for the exchange‐correlation potential.^[^ ^66^, ^67^ ^]^ The long‐range van der Waals interaction was described by the DFT‐D3 approach.^[^ ^68^ ^]^ The cut‐off energy for the plane wave was set to 480 eV. The energy criterion was set to 10^−5^ eV in the iterative solution of the Kohn–Sham equation. All the structures were relaxed until the residual forces on the atoms declined to less than 0.02 eV Å^−1^. Data analysis and visualization were carried out with the help of VASPKIT^[^ ^69^ ^]^ code and VESTA.^[^ ^70^ ^]^ Here, Δρ  = ρ_A + B_  − ρ_A_ − ρ_B_ was defined as the charge density difference of A/B heterostructure, where ρ_A/B_, ρ_A _and ρ_B _are the charge densities of A/B heterostructure, isolated A and B slabs, respectively. Te Bader charge to express the charge transfer quantity was used. The adsorption energy Eads is expressed as

(11) $$\Delta E\mathrm{ads}\;=E_{A+B}\;-E_{A}-E_{B}$$

where *E*_A + B_ is the total energy of slab A model with B adsorption, *E*_A_ is the energy of a A slab, and *E*_B_ is that for a B molecule.

Here, differences in Gibbs free energy (*ΔG*) for intermediates defined as:

(12) $$\Delta G=\Delta E+\Delta E_{ZPE}-T\Delta S+\Delta G_{U}$$

where *ΔG* is the total energy difference between the slab and respective terminations computed by DFT‐PBE. *ΔE*_ZPE_ and *TΔS* denote differences in zero‐point energy and entropy between adsorbed states of reaction intermediates and gap phase, respectively. *T* is the room temperature (298.15 K). *ΔGU* = −eU, whereby *U* is the electrode potential.

## Conflict of Interest

The authors declare no conflict of interest.

## Author Contributions

B.‐T.L. and X.L. conceived the idea and designed the experiments. B.‐T.L., X.Z., L.Z., J.C., and Z.L. carried out the fabrication of materials and performed the electrochemical and microstructural characterizations. J.Z., Y.W. and Z.X. contributed to the DFT calculation. B.‐T.L., L.Z. and X.Z. wrote the paper. X.L. and T.M. revised the paper. All authors discussed the results and commented on the manuscript.

## Supporting information



Supporting Information

## Data Availability

The data that support the findings of this study are available from the corresponding author upon reasonable request.
