# Supplementary material for: Solar Trap‐Adsorption Photocathode for Highly Stable 2.4 V Dual‐Ion Solid‐State Iodine Batteries
Source: Adv Mater. 2025 Aug 6;37(42):e04492. doi: 10.1002/adma.202504492 (PMC12548518; doi:10.1002/adma.202504492)
Supplement: Supplementary file 1 — Supporting Information [file ADMA-37-e04492-s001.docx]

Supporting Information

Solar Trap-Adsorption Photocathode for Highly Stable 2.4 V Dual-Ion Solid-State Iodine Batteries

Xueying Zhang^a^, Lingfeng Zhu^b^*, Jiale Cao^a^, Zheng Li^a^, Youliang Wang^c^, Jianwei Zhao^d^, Zhencheng Xie^b^, Xiaoning Li^b^*, Tianyi Ma^b^, and Bo-Tian Liu^a^*

^a^ Guangxi Key Laboratory of Electrochemical and Magneto-chemical Functional Materials, Guilin University of Technology, Guilin 541004, China

^b^ Centre for Atomaterials and Nanomanufacturing (CAN), School of Science, RMIT University, Melbourne, VIC 3000, Australia

^c^ School of Chemistry and Chemical Engineering, Nanchang University, Nanchang, 330031, China

^d^ Shenzhen HUASUAN Technology Co., Ltd. Shenzhen, P. R. China

* Corresponding authors:

Email: btliu2018@glut.edu.cn (B. Liu); xiaoning.li@rmit.edu.au (X. Li); lingfeng.zhu@rmit.edu.au (L. Zhu).


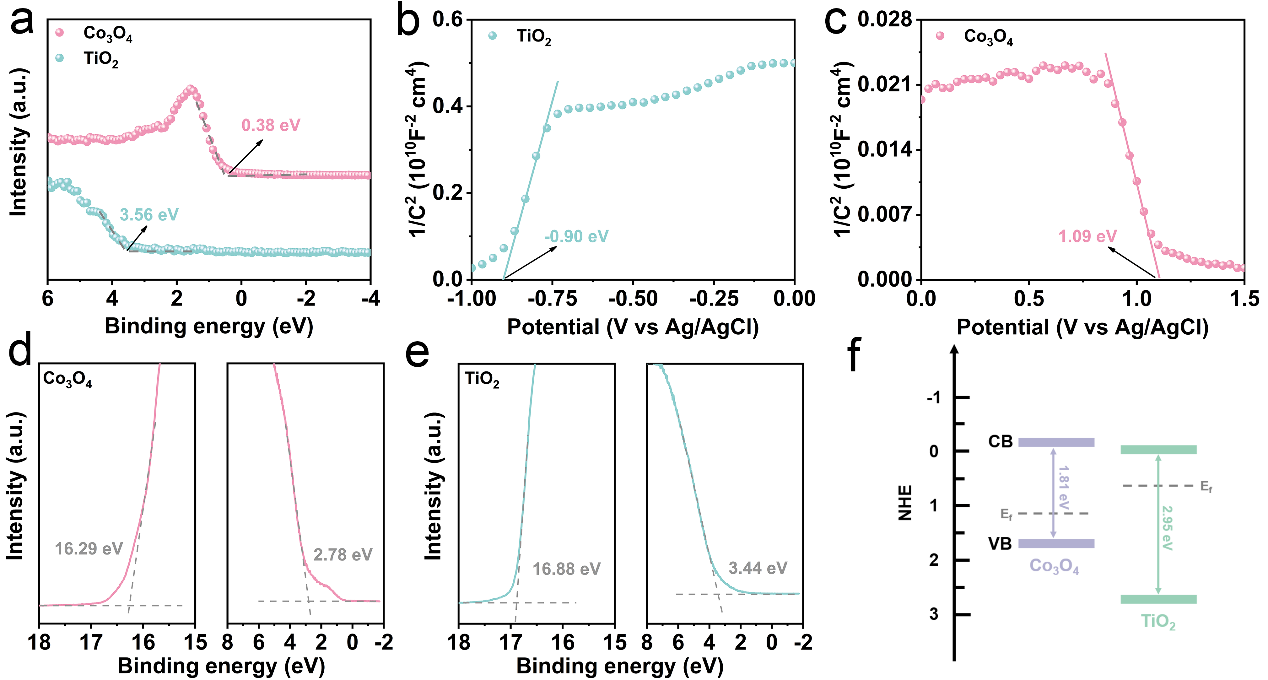


**Figure S1**. (a) VB-XPS spectra of TiO_2_ and Co_3_O_4_; Mott-Schottky plots of (b) TiO_2_ and (c) Co_3_O_4_; Ultraviolet photoelectron spectroscopy of (d) Co_3_O_4_ and (e) TiO_2_; (f) Schematic band structures of Co_3_O_4_ and TiO_2_.


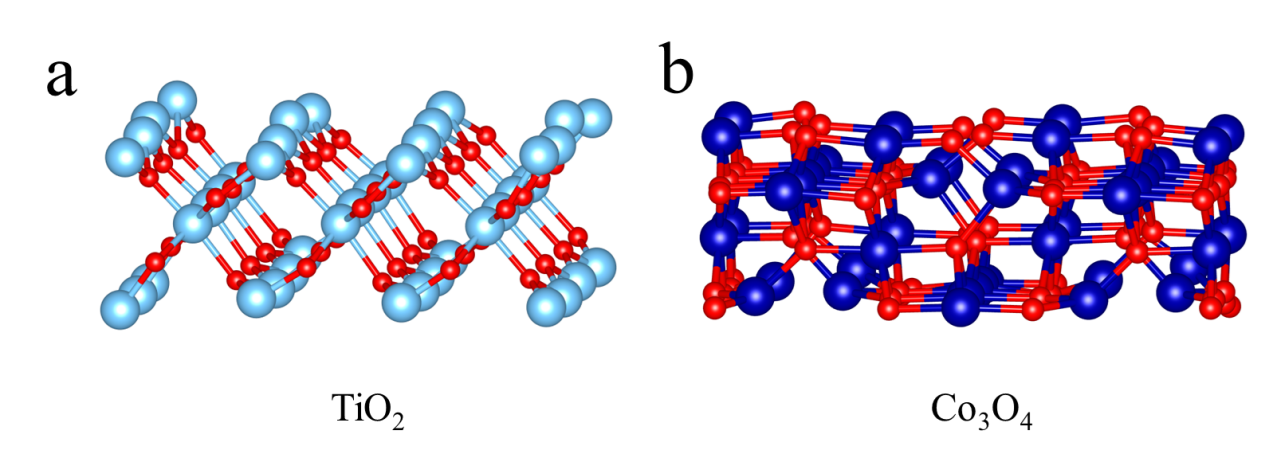


**Figure S2**. Crystal structure of (a) TiO_2_ and (b) Co_3_O_4_.


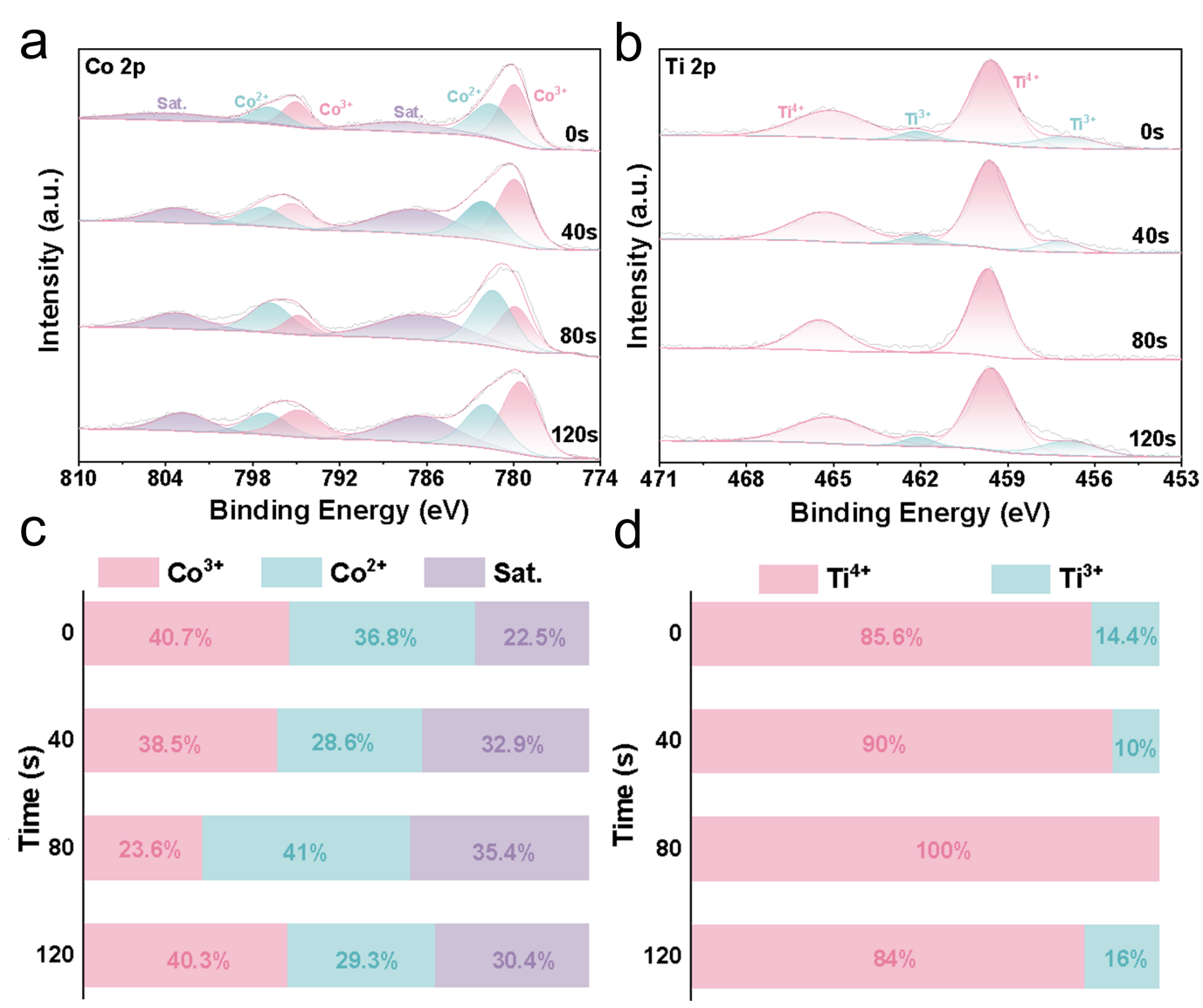


Figure S3. High-resolution XPS depth profiling and elemental composition analysis of the Co_3_O_4_-TiO_2_/CC photocathode. (a) Co 2p; and (b) Ti 2p spectra obtained at various sputtering times (pristine, 40s, 80s, and 120s) using an Ar cluster ion beam sputtering; (c, d) Percentage statistics of the corresponding elemental components.


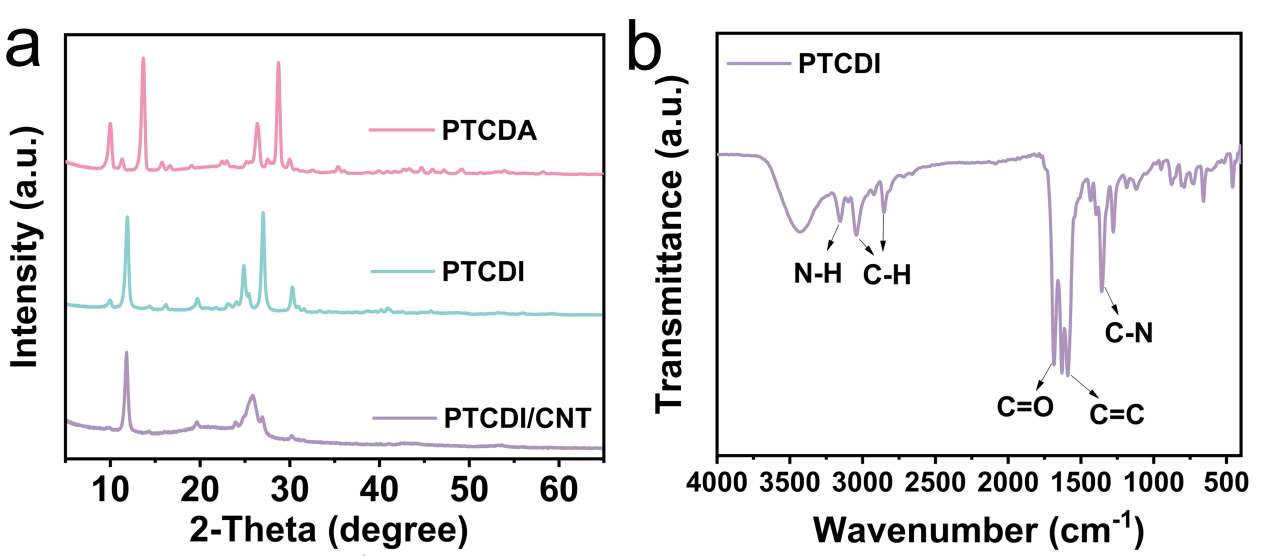


**Figure S4**. (a) XRD patterns and FT-IR spectra; (b) FTIR of PTCDI/CNT samples.


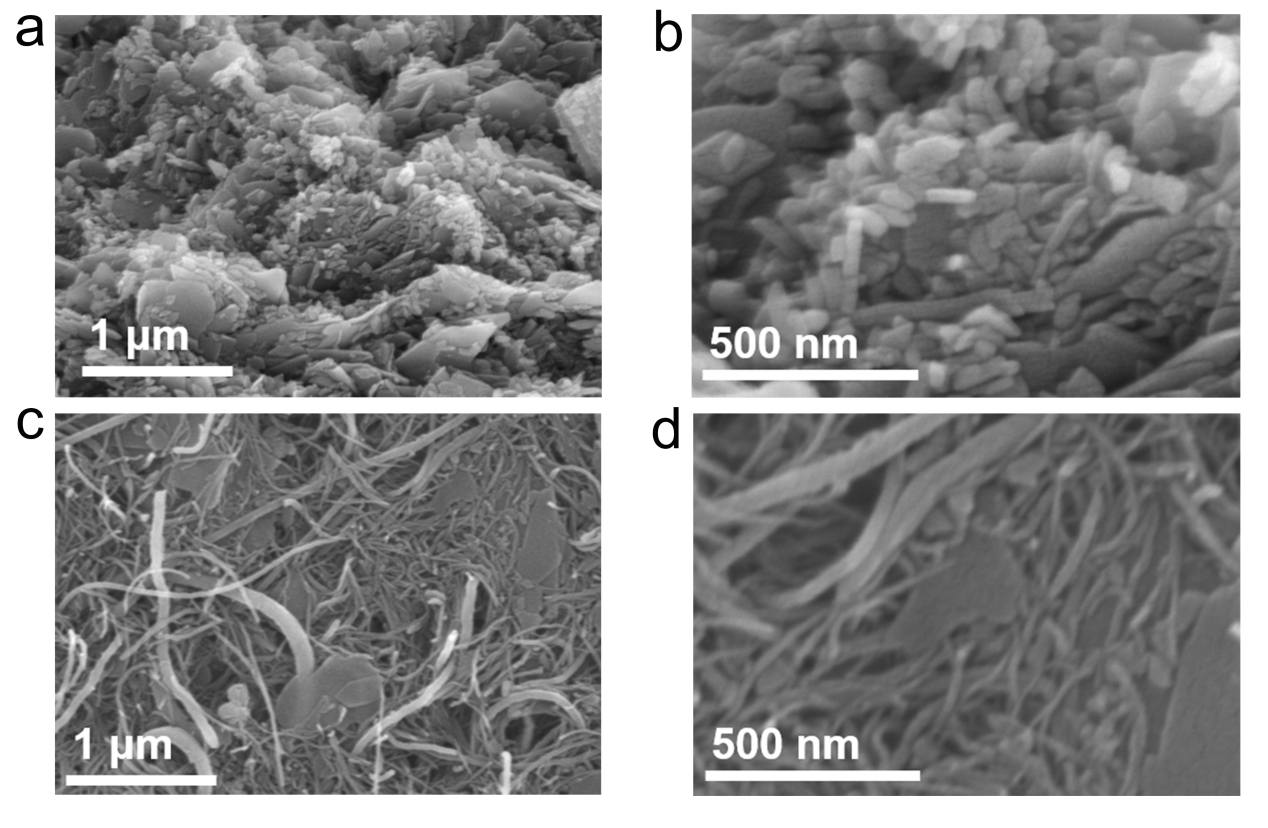


**Figure S5**. Low-magnification SEM image of (a) PTCDI and (c) PTCDI/CNT anode; High-magnification SEM image of (b) PTCDI and (d) PTCDI/CNT anode.


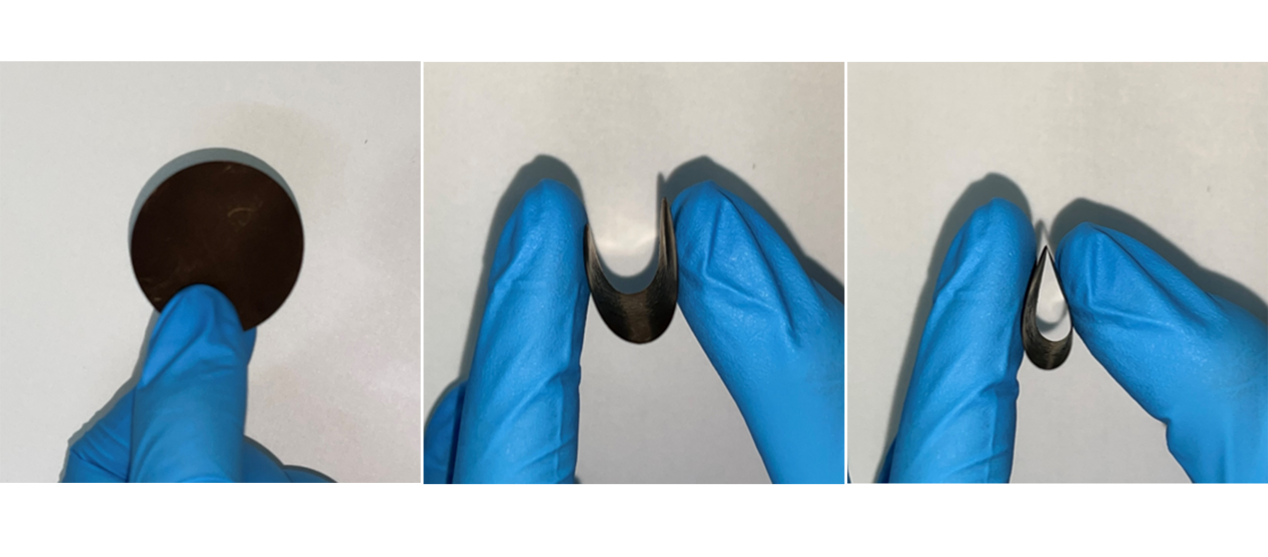


**Figure S6**. Optical photographs of PTCDI/CNT anode bended from 0 to 180°.


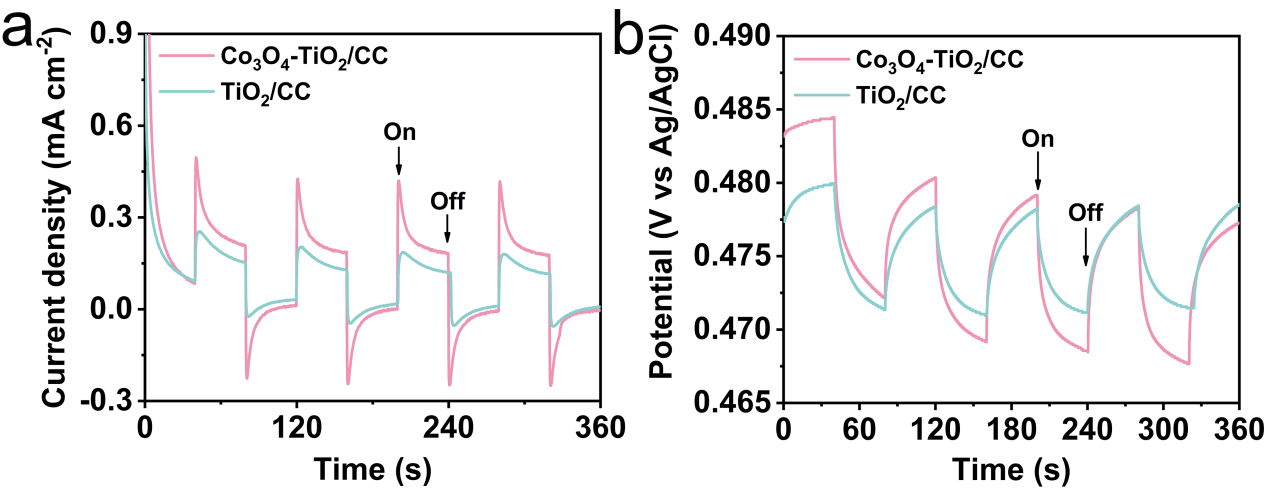


**Figure S7**. (a) Photocurrent response spectra of Co_3_O_4_-TiO_2_/CC photocathode and TiO_2_/CC photocathode in aqueous electrolyte; (b) Open circuit potential curves of electrodes under intermittent illumination.


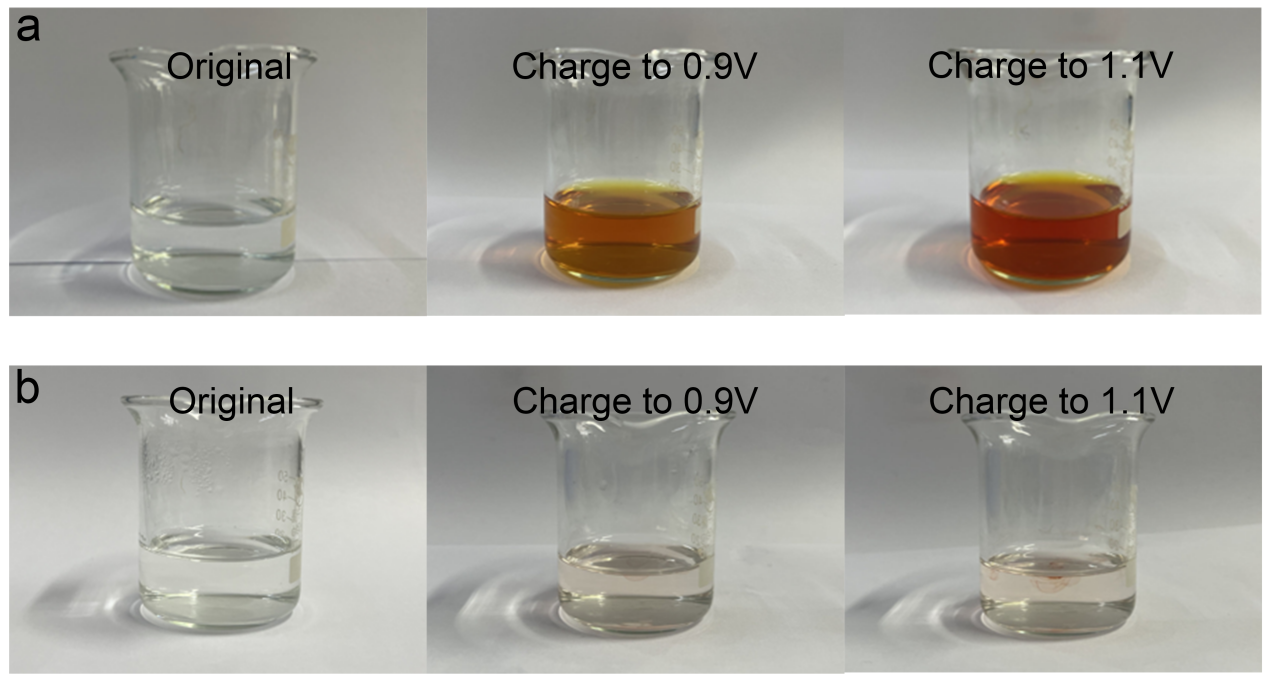


**Figure S8**. Optical photographs of (a) aqueous electrolyte and (b) gel electrolyte in different charged states with a scan rate of 5 mV s^-1^.


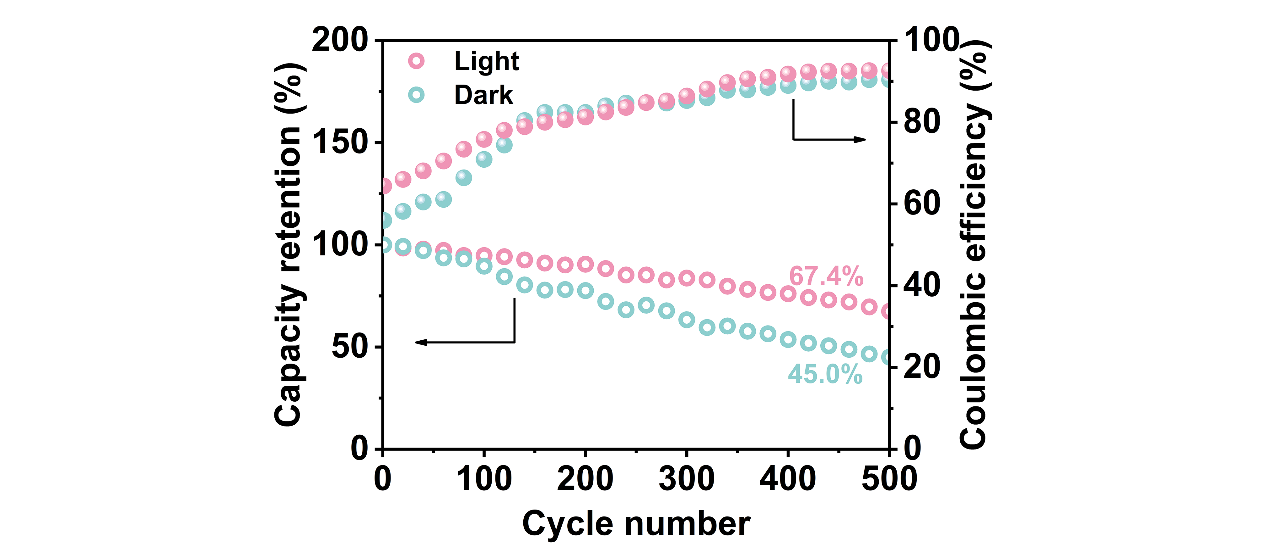


**Figure S9**. Cycle performance of Co_3_O_4_-TiO_2_/CC photocathode in aqueous electrolyte at 10 mA cm^-2^ current density comparing light and dark conditions.


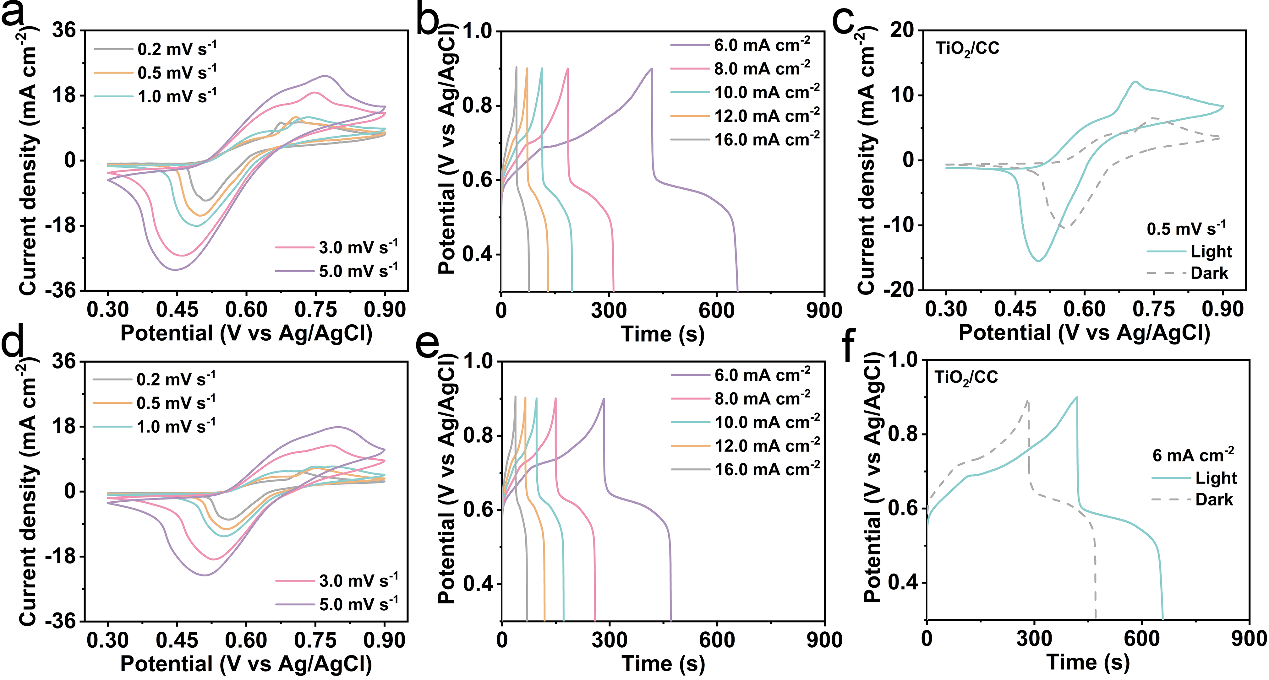


**Figure S10**. Electrochemical performance of TiO_2_/CC photocathode in aqueous electrolyte. (a) CV curves of TiO_2_/CC photo-cathode at various scan rates under illumination; (b) GCD profiles of TiO_2_/CC photo-cathode at various current densities under illumination; (c) Comparative CV curves of TiO_2_/CC photo-cathode at a scan rate of 0.5 mV s^-1^ under illumination and dark condition; (d) CV curves of TiO_2_/CC photo-cathode at various scan rates under dark condition; (e) GCD profiles of TiO_2_/CC photo-cathode at various current densities under dark condition; (f) Comparative CV curves of TiO_2_/CC photo-cathode at a current density of 6 mA cm^-2^ under illumination and dark condition.


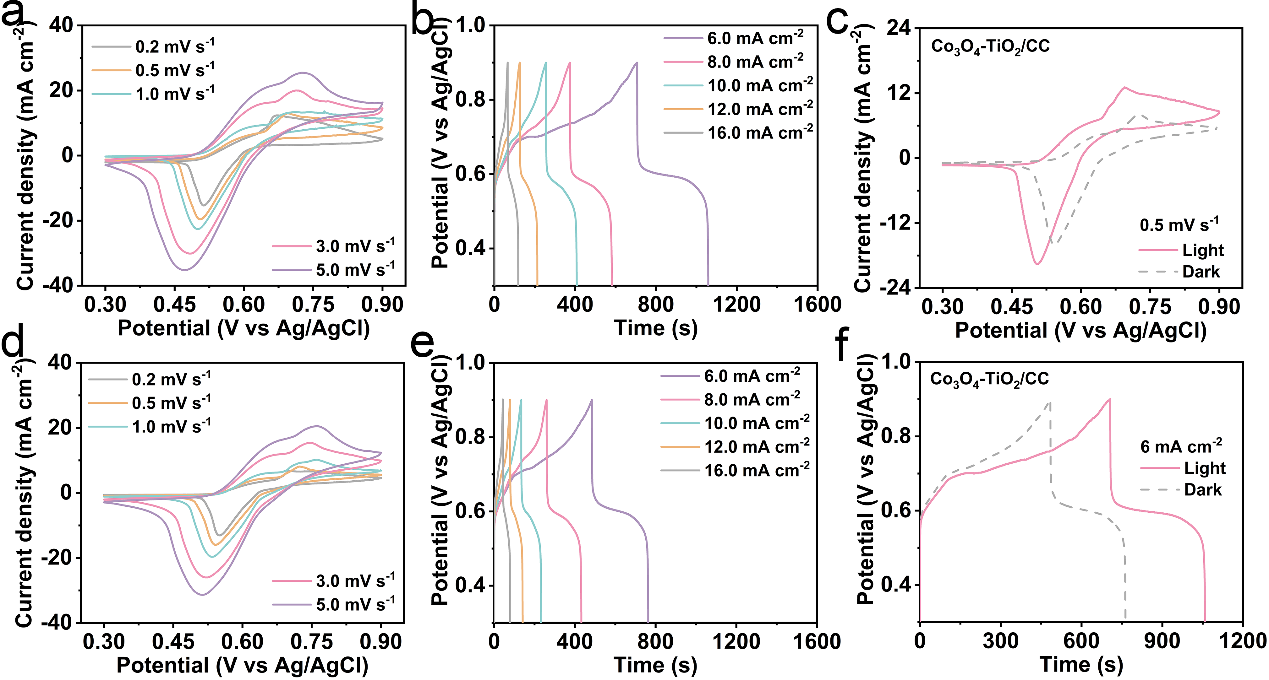


**Figure S11**. Electrochemical performance of Co_3_O_4_-TiO_2_/CC photocathode in aqueous electrolyte. (a) CV curves of Co_3_O_4_-TiO_2_/CC photo-cathode at various scan rates under illumination; (b) GCD profiles of Co_3_O_4_-TiO_2_/CC photo-cathode at various current densities under illumination; (c) Comparative CV curves of Co_3_O_4_-TiO_2_/CC photo-cathode at a scan rate of 0.5 mV s^-1^ under illumination and dark condition; (d) CV curves of Co_3_O_4_-TiO_2_/CC photo-cathode at various scan rates under dark condition; (e) GCD profiles of Co_3_O_4_-TiO_2_/CC photo-cathode at various current densities under dark condition; (f) Comparative CV curves of Co_3_O_4_-TiO_2_/CC photo-cathode at a current density of 6 mA cm^-2^ under illumination and dark condition.


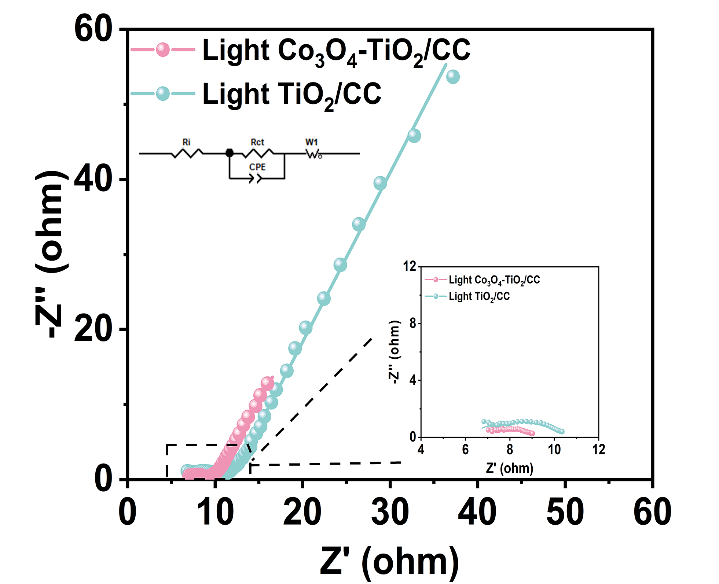


**Figure S12**. EIS spectra of Co_3_O_4_-TiO_2_/CC photocathode and TiO_2_/CC photocathode.





**Figure S13**. Comparison of specific capacities of Co_3_O_4_-TiO_2_/CC, TiO_2_ and Co_3_O_4_ at a scan rate of 0.2 mV s^-1^.


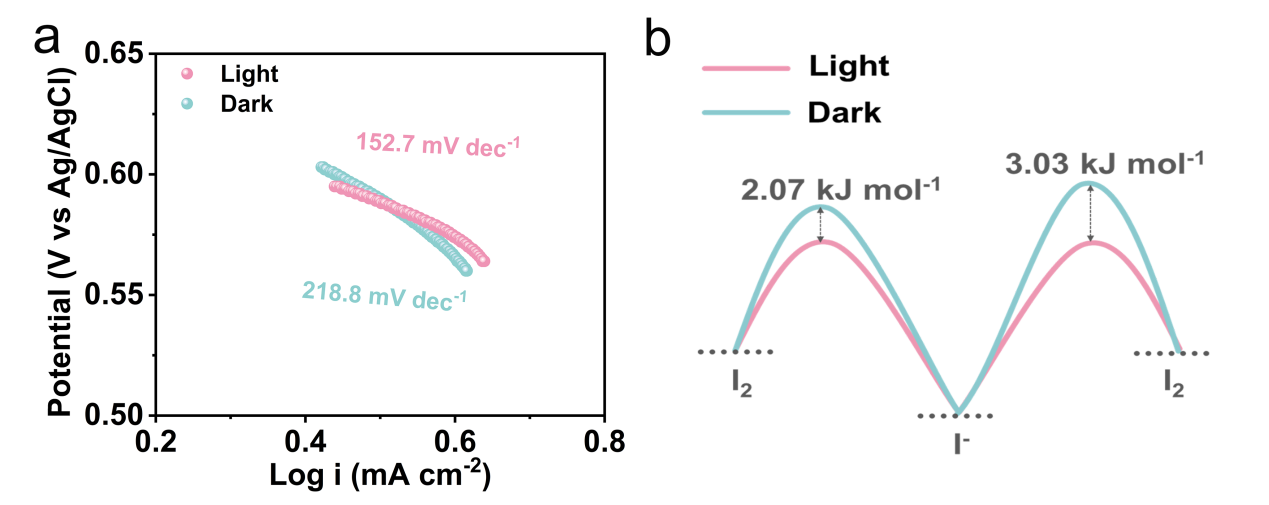


**Figure S14**. (a) Tafel slopes of Co_3_O_4_-TiO_2_/CC cathode under light and dark conditions during the reduction process; (b) Comparison of the activation energies of Co_3_O_4_-TiO_2_/CC cathode in the oxidation and reduction processes with and without illumination, derived from electrochemical parameters.


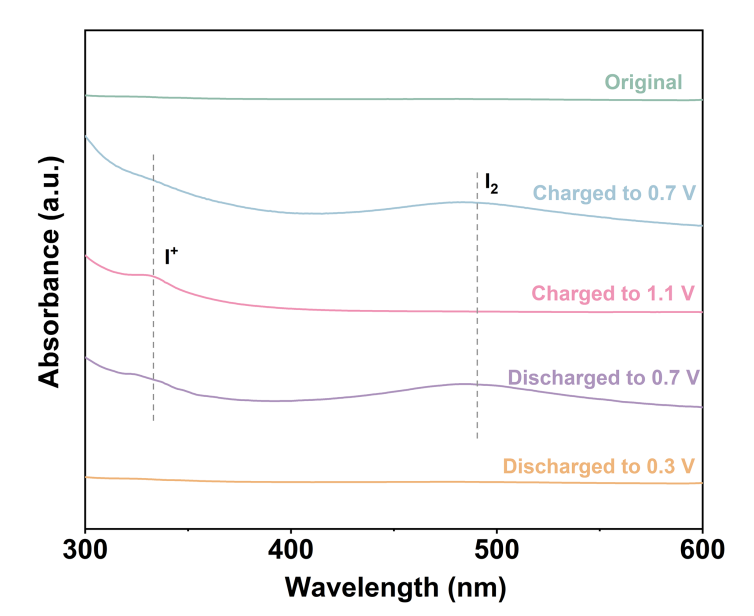


**Figure S15**. Ex-situ UV-vis absorption spectroscopy of the Co_3_O_4_-TiO_2_/CC cathode recorded at various charge/discharge stages.


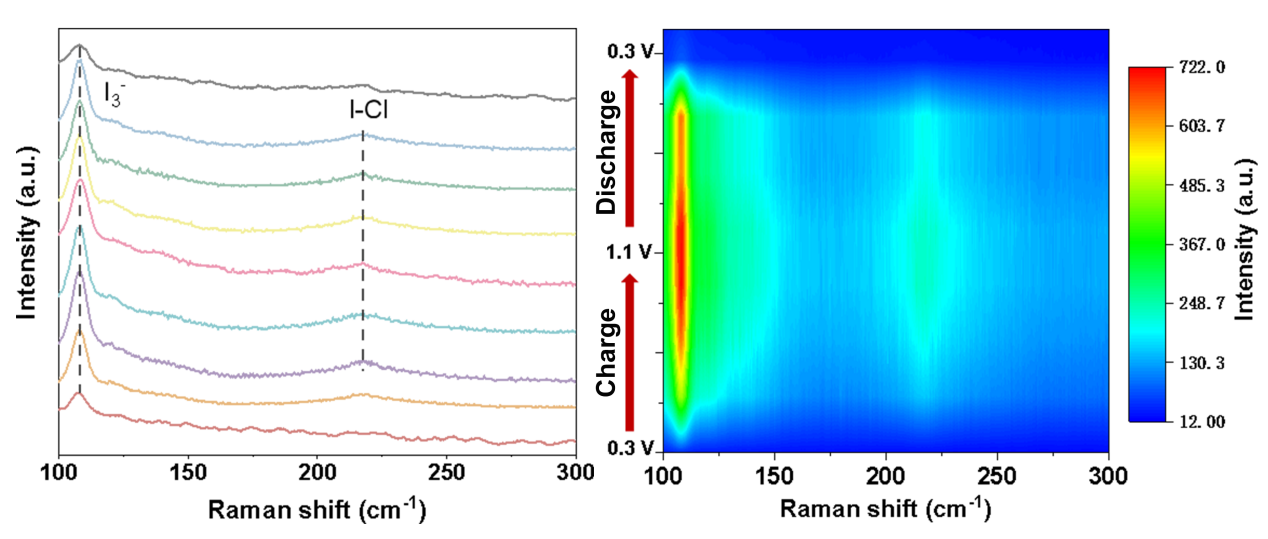


**Figure 16.** In-situ Raman spectra of Co_3_O_4_-TiO_2_/CC photo-assisted cathode during charge/discharge processes.


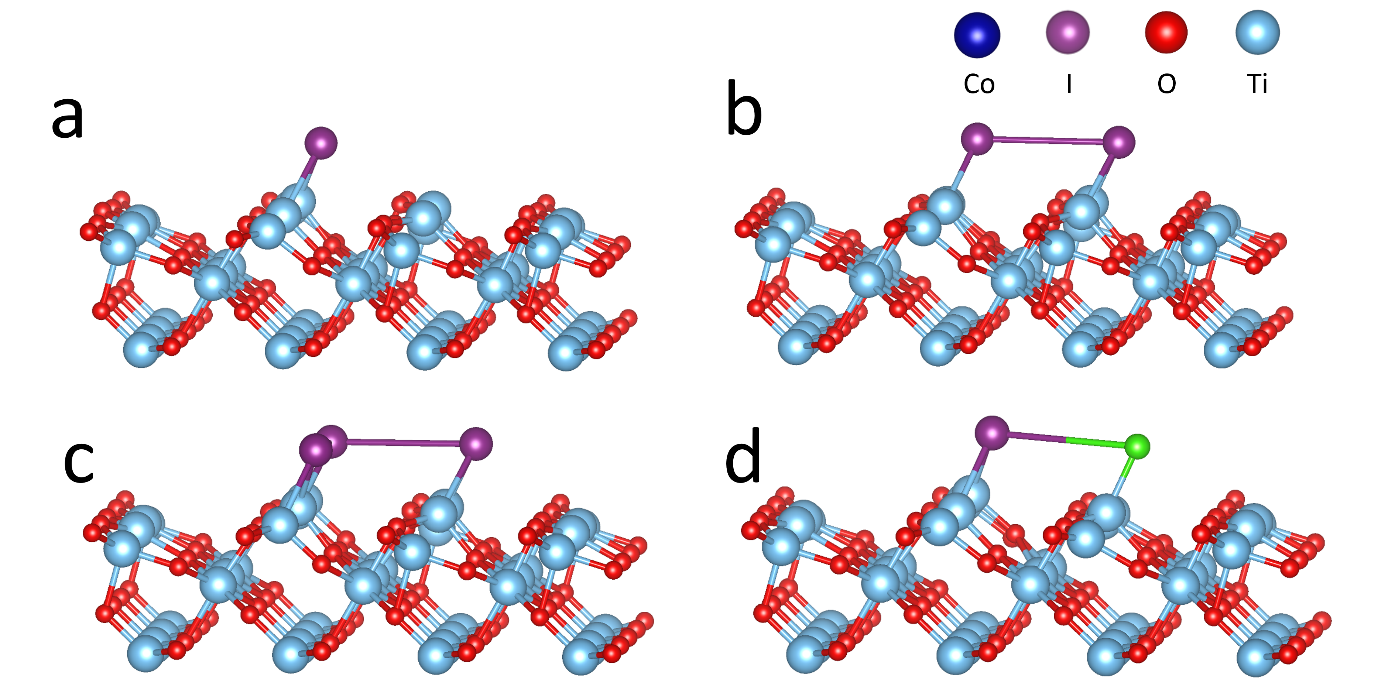


**Figure S17.** The corresponding adsorption configuration calculated by DFT. (a) I^-^, (b) I_2_, (c) I_3_^-^ and (d) ICl on the TiO_2_/CC surface, respectively.


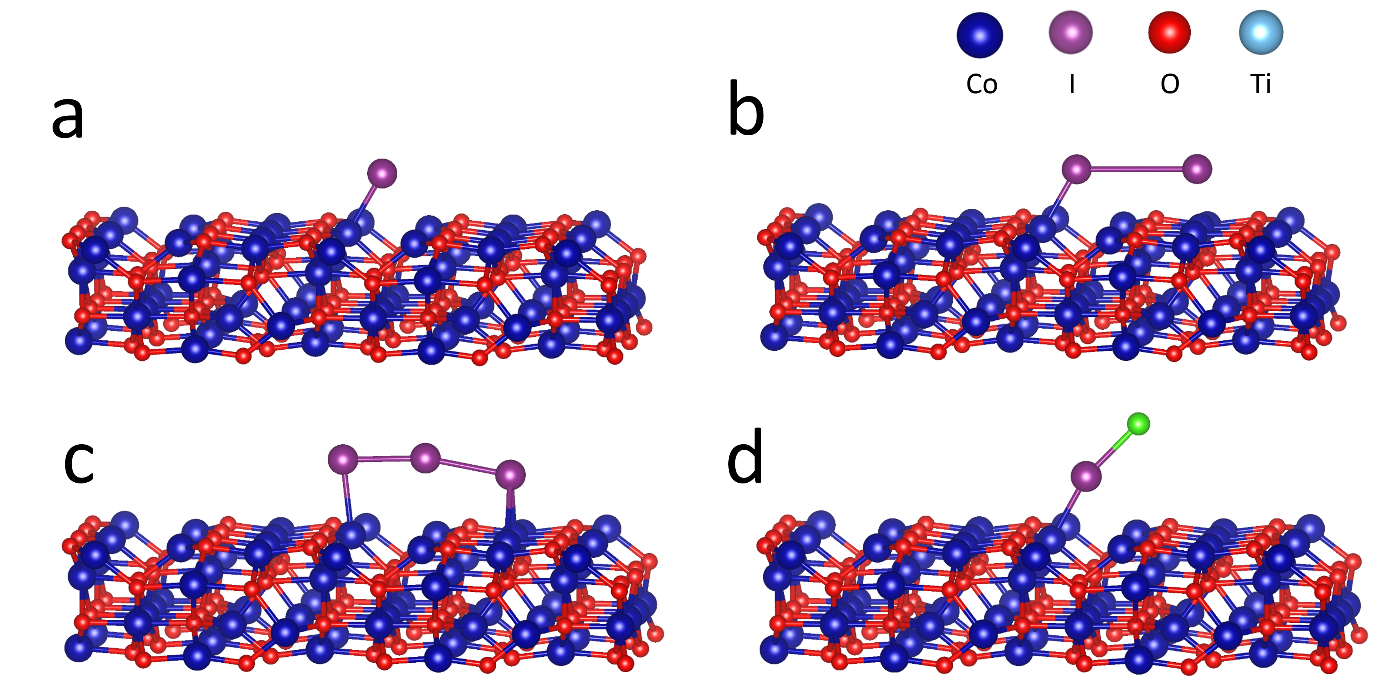


**Figure S18.** The corresponding adsorption configuration calculated by DFT. (a) I^-^, (b) I_2_, (c) I_3_^-^ and (d) ICl on the Co_3_O_4_/CC surface, respectively.


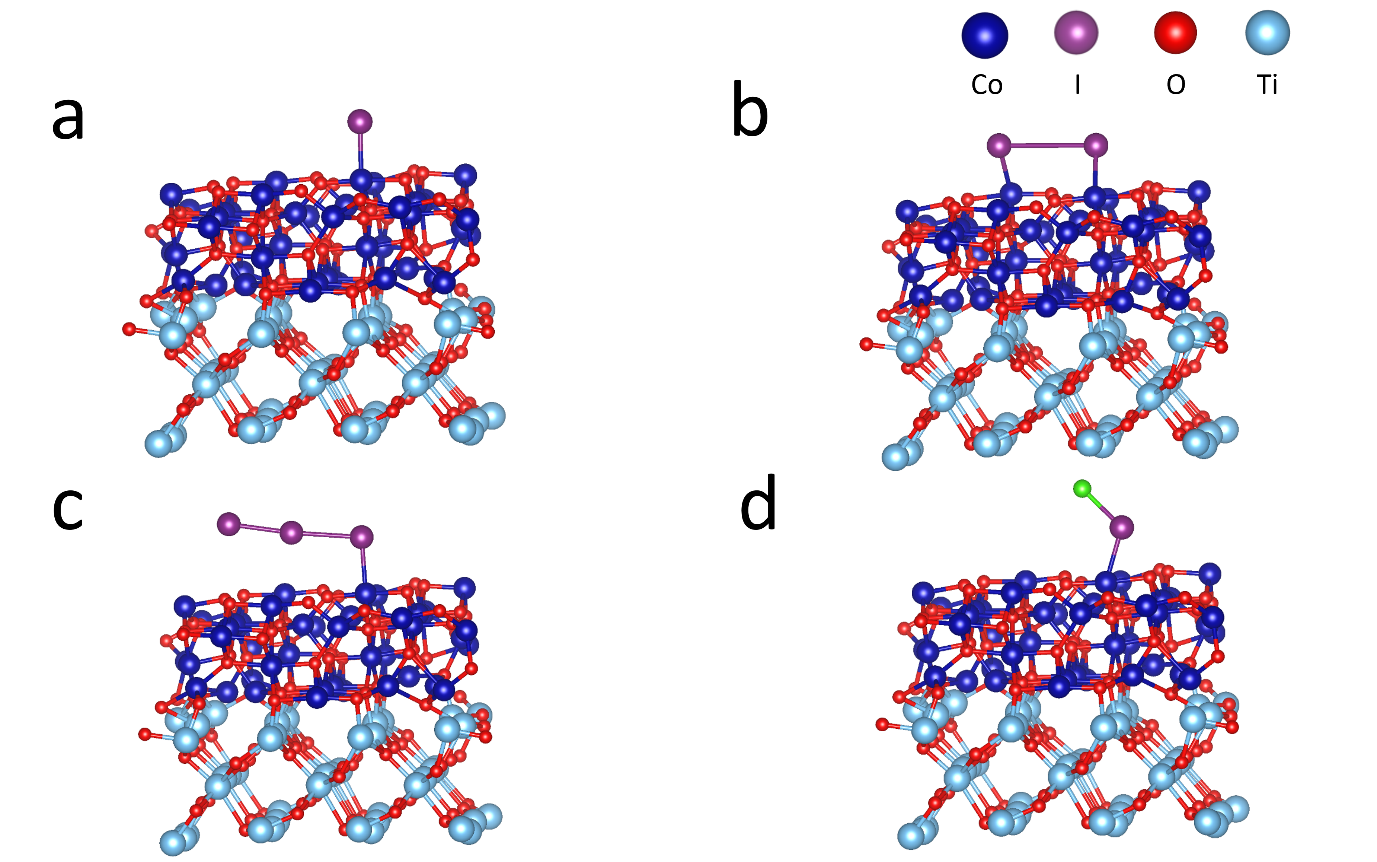


**Figure S19.** The corresponding adsorption configuration calculated by DFT. (a) I^-^, (b) I_2_, (c) I_3_^-^ and (d) ICl on the Co_3_O_4_-TiO_2_/CC surface, respectively.

**
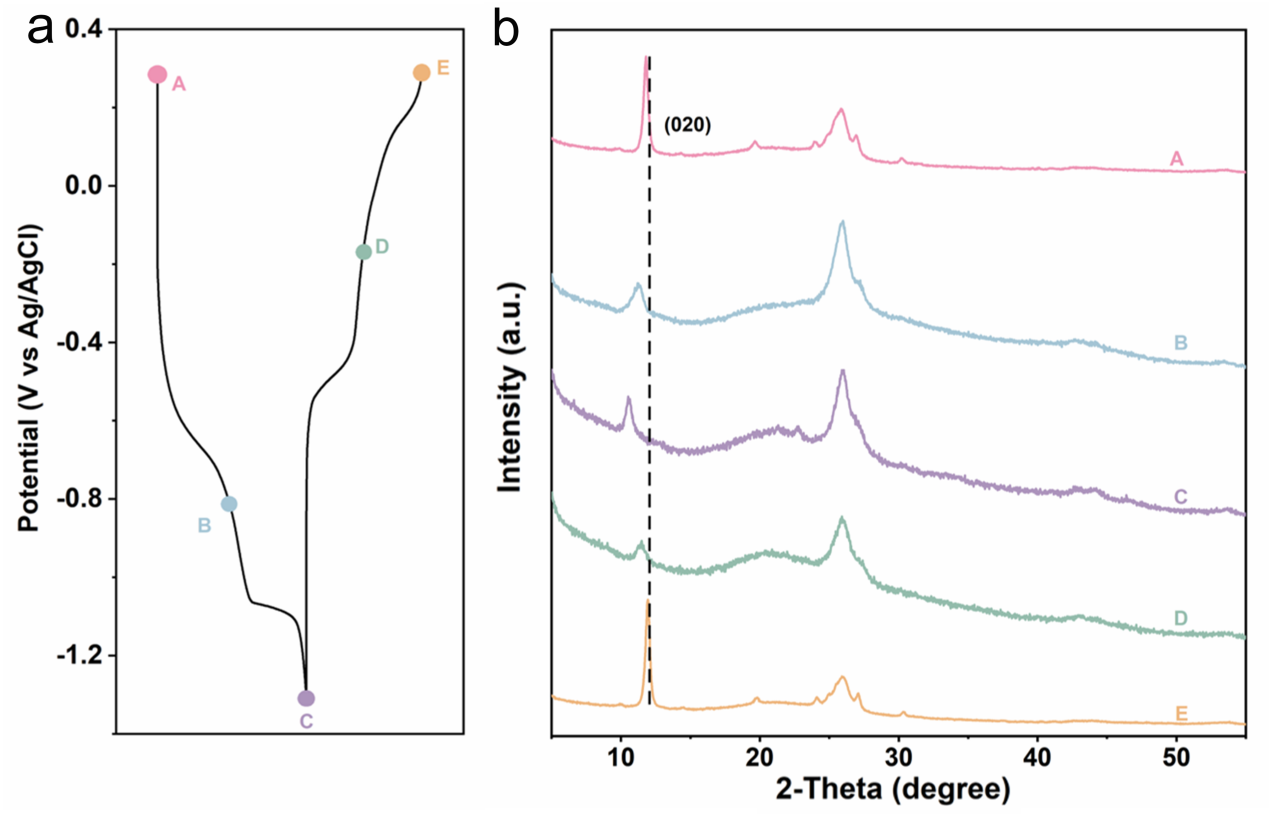
**

**Figure S20.** (a) Typical galvanostatic charge/discharge profiles of the PTCDI/CNT anode at selected states of charge; (b) Ex-situ XRD patterns of the PTCDI/CNT anode at different state of charge.


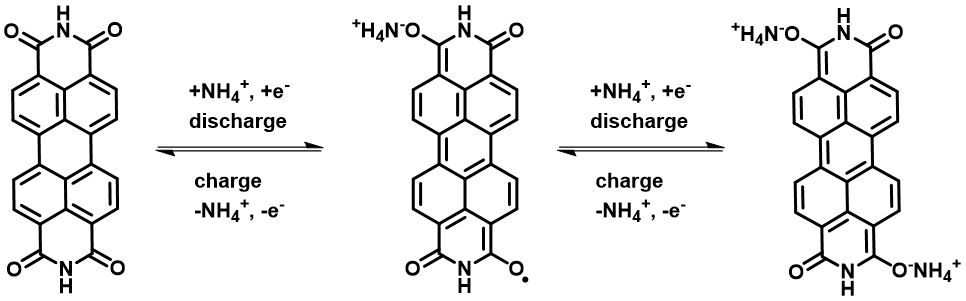


**Figure S21.** Redox mechanism of PTCDI for NH_4_^+^ cation incorporation.


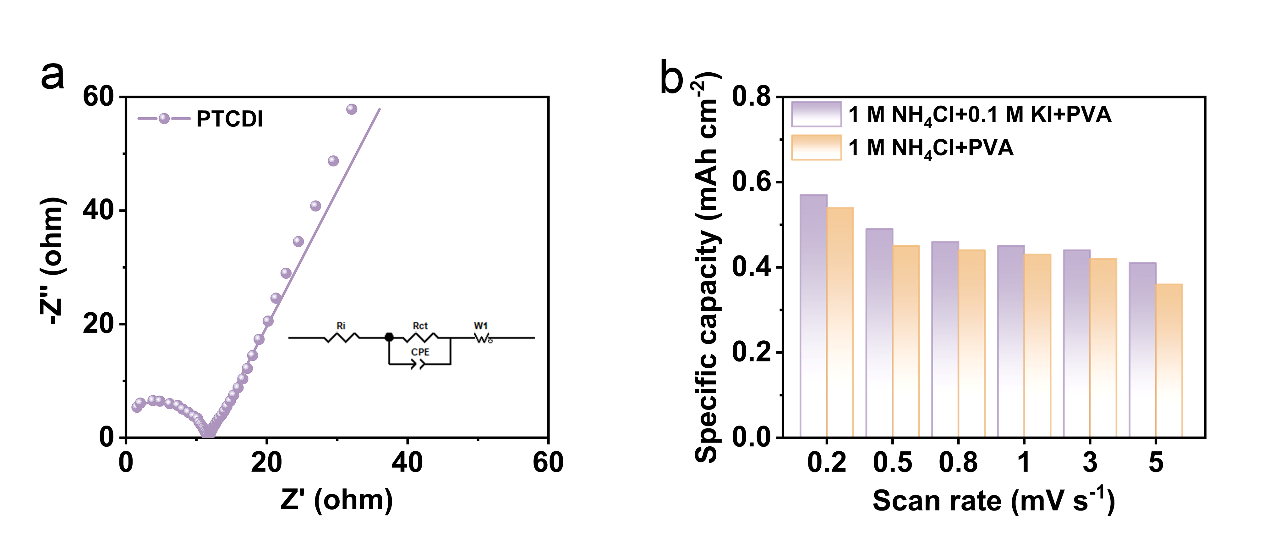


**Figure S22**. (a) EIS spectra of PTCDI/CNT anode; (b) Comparative specific capacity of PTCDI/CNT anode at different electrolyte.


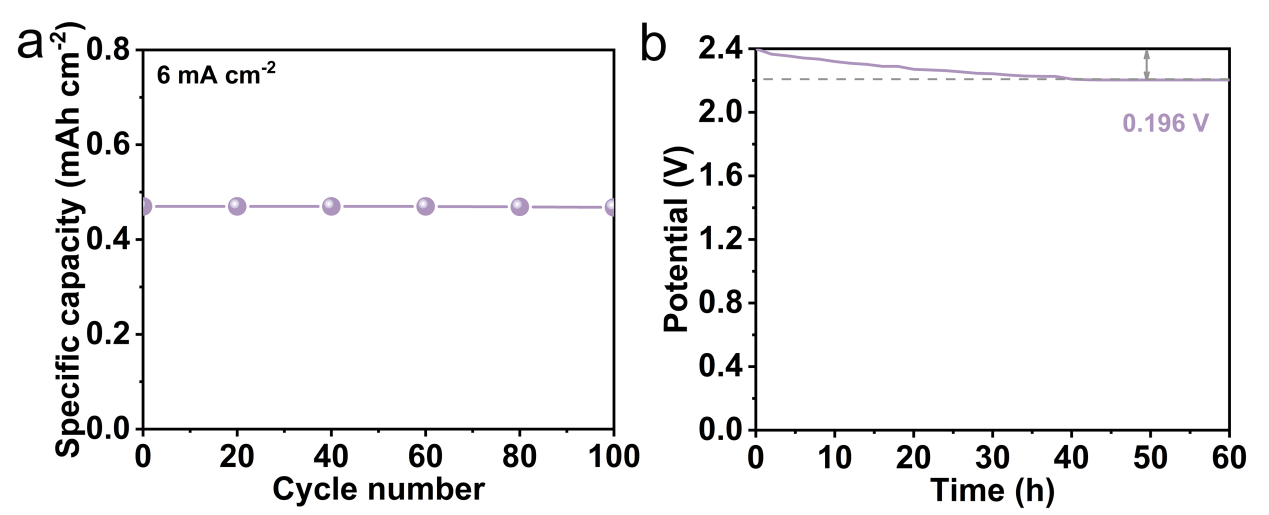


**Figure S23.** (a) Cycle performance of solid-state PTCDI||I_2_ batteries at current density of 6 mA cm^-2^; (b) Self-discharge curves of solid-state PTCDI||I_2_ batteries.


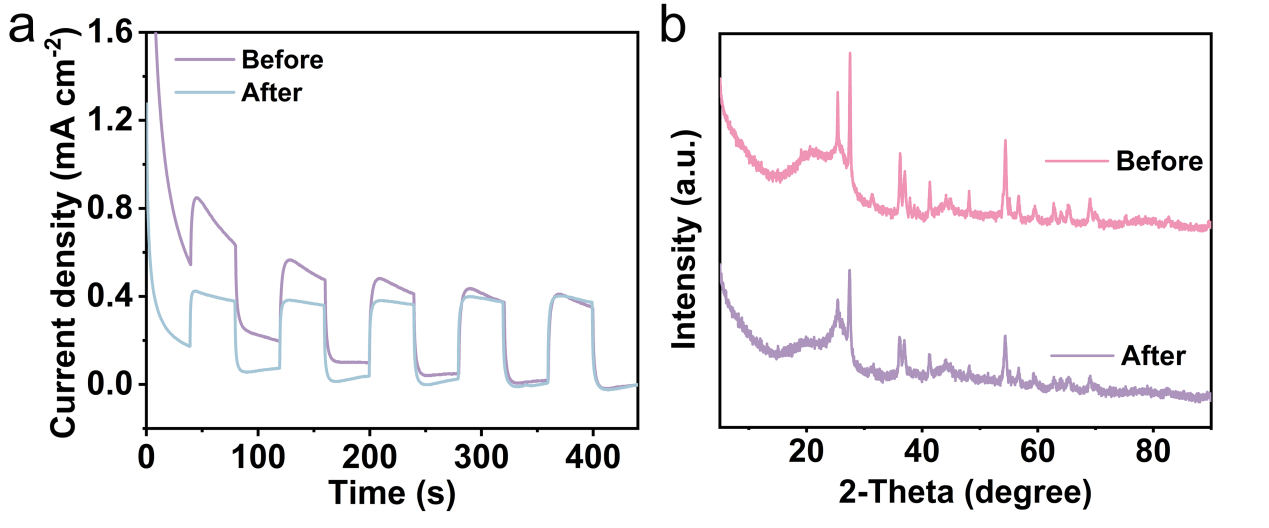


**Figure S24.** (a) Photocurrent response spectra; (b) XRD pattern of the Co_3_O_4_-TiO_2_/CC before and after 1000 cycles.


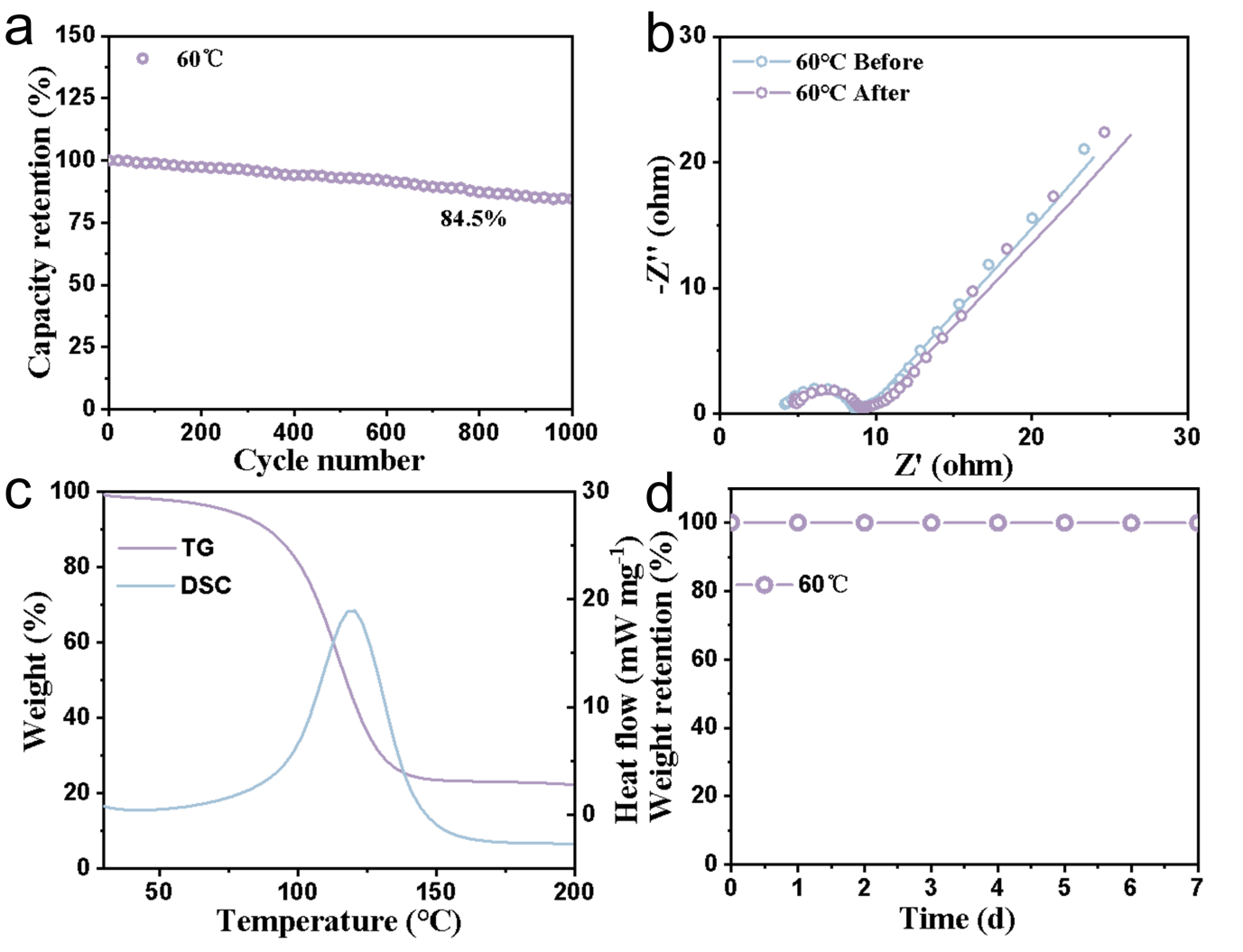


**Figure S25.** (a) Cycle performance of solid-state PTCDI||I_2_ batteries at a current density of 10 mA cm^-2^; (b) Comparison of the EIS spectra before and after 1000 cycling at 60 ℃; (c) TG-DCS curve of PVA-based gel electrolyte; (d) Weight retention of PVA-based gel electrolyte after seven days of static storage at 60 ℃.


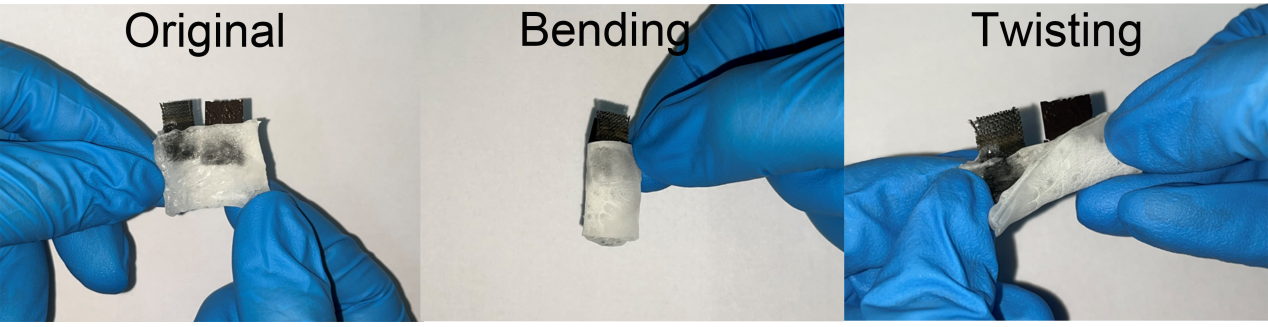


**Figure S26**. Digital photos of solid-state PTCDI||I_2_ prototype batteries bent and twisted from 0 to 180°.

**Table S1.** Comparison in specific capacity of Co_3_O_4_-TiO_2_/CC electrode under different illumination conditions

| **Light intensity** | **Specific capacity (mAh cm^-2^)** |
| --- | --- |
| 100% | 1.24 |
| 50% | 1.07 |
| 10% | 0.92 |
| 0% | 0.87 |

| **Cathode** | **Electrolyte** | **Anion** | **Active** **mass loading (mg cm^-2^)** | **Current density**  **(A g^-1^)** | **Specific** **Capacity (mAh cm^-2^)** | **Ref.** |
| --- | --- | --- | --- | --- | --- | --- |
| BFO@C | 1M KF+3M KOAc | F^-^ | 1.2~1.5 | 0.1 | 0.47 | ^[1]^ |
| Graphite | 9M MgCl_2_+30M ChCl | Cl^-^ | 2.5 | 0.1 | 0.38 | ^[2]^ |
| PC/AC | 1M TPABr + 9.1M LiBr | Br^-^ | 1 | 0.5 | 0.54 | ^[3]^ |
| PBI | 1M Na_2_SO_4_+0.1M H_2_SO_4_+2.5mM KI | I^-^ | 0.5~2 | 1 | 0.22 | ^[4]^ |
| I_2_@AC | 1M CuSO_4_+0.5M CuCl_2_ | ICl | 1 | 1 | 0.56 | ^[5]^ |
| **Co_3_O_4_-TiO_2_/CC** | **1M NH_4_Cl+0.1M KI** | **I^-^/ICl** | **4.5** | **0.2mV s^-1^** | **1.24/1.43**  **(Light)** | **This work** |

**Table S2**. Comparison in specific capacity of Co_3_O_4_-TiO_2_/CC electrode with other halogens-based batteries.

**Table S3**. Comparison of adsorption energies of iodine species on the surface of various models.

| **Models** | **Adsorption energies (eV)** |
| --- | --- |
| TiO_2_/CC-I^-^ | -0.88 |
| TiO_2_/CC-I_2_ | -1.33 |
| TiO_2_/CC-I_3_^-^ | -1.84 |
| TiO_2_/CC-ICl | -1.06 |
| Co_3_O_4_/CC-I^-^ | -1.09 |
| Co_3_O_4_/CC-I_2_ | -1.64 |
| Co_3_O_4_/CC-I_3_^-^ | -1.91 |
| Co_3_O_4_/CC-ICl | -1.19 |
| **Co_3_O_4_-TiO_2_/CC-I^-^** | **-2.40** |
| **Co_3_O_4_-TiO_2_/CC-I_2_** | **-2.58** |
| **Co_3_O_4_-TiO_2_/CC-I_3_^-^** | **-2.99** |
| **Co_3_O_4_-TiO_2_/CC-ICl** | **-1.49** |

**Table S4**. Comparison of maximum power and energy density of solid-state PTCDI||I_2_ prototype batteries with various previously reported storage devices.

| **Cathode** | **Anode** | **Power density (mW cm^-2^)** | **Energy density (mWh cm^-2^)** | **Ref.** |
| --- | --- | --- | --- | --- |
| PBI | Zn | 0.24 | 0.29 | [4] |
| I_2_@GP-CMT | Zn/CB | 1.93 | 0.27 | [7] |
| Ni-APW | PTCDI | 0.54 | 0.37 | [8] |
| MnAl LDH | PTCDI | 0.49 | 0.28 | [9] |
| MnO_2_ | WO_3_ | 1 | 0.21 | [10] |
| Na_0.6_MnO_2_ | d-V_2_CTX | 1 | 0.08 | [11] |
| V_2_O_5_ | V_2_O_5_ | 0.19 | 0.17 | [12] |
| FeHCF | PTCDI | 0.1 | 0.06 | [13] |
| IBr | Zn | 0.28 | 0.36 | [14] |
| Br/FeSAC-CMK | Zn | 0.14 | 0.24 | [15] |
| **Co_3_O_4_-TiO_2_/CC** | **PTCDI/CNT** | **7.33** | **0.66** | **This work** |

**Table S5**. Comparison in electrochemical properties of PTCDI||I_2_ batteries with halogens and ammonium-based full cells.

| **Cathode** | **Anode** | **Potential window (V)** | **Current density**  **(C/A g^-1^)** | **Cycle number** | **Ref.** |
| --- | --- | --- | --- | --- | --- |
| BFO@C | Zn | 0-1.2 | 1 | 1500 | ^[1]^ |
| PC/AC | NDPI | 0-1.7 | 1 | 1000 | ^[3]^ |
| PBI | Zn | 0.5-1.7 | 4 | 1500 | ^[4]^ |
| I_2_@AC | Zn | 1.3-1.8 | 4 | 50 | ^[5]^ |
| Ag | BiOCl | 0-1.5 | 0.4 | 45 | ^[6]^ |
| I_2_@GP-CMT | Zn/CB | 1.2-1.8 | 4 | 1000 | ^[7]^ |
| Ni-APW | PTCDI | 0-1.8 | 3C(1C=40mA g^-1^) | 1000 | ^[8]^ |
| MnAl LDH | PTCDI | 0-1.6 | 0.1 | 100 | ^[9]^ |
| MnO_2_ | WO_3_ | 0-1.7 | 3 | 5000 | ^[10]^ |
| Na_0.6_MnO_2_ | d-V_2_CT_X_ | 0-1.6 | 1 | 500 | ^[11]^ |
| **Co_3_O_4_-TiO_2_/CC** | **PTCDI/CNT** | **0-2.4** | **10mA cm^-2^** | **1000** | **This work** |

**Table S6**. Comparison of photoconversion efficiencies among various photo-assisted battery systems.

| **Photoelectrode** | **Photo conversion efficiency (%)**  **η_photoassisted_ =** | **Ref.** |
| --- | --- | --- |
| Ni/CdS@Ni_3_S_2_ | 0.11 | ^[16]^ |
| Cs_3_Bi_2_I_9_ | 0.43 | ^[17]^ |
| rGO/CdS | 5.04 | ^[18]^ |
| α-MnO_2_ | 1.2 | ^[19]^ |
| CdS-TiO_2_ | 2.3 | ^[20]^ |
| Cu/CuO | 0.21 | ^[21]^ |
| **Co_3_O_4_-TiO_2_** | **3.21** | **This work** |

**References**

[1] H. Wang, C. Lei, T. Liu, C. Xu, X. He, X. Liang, *Angew. Chem. Int. Ed.* **2024**, *136*, e202401483.

[2] K. i. Kim, Q. Guo, L. Tang, L. Zhu, C. Pan, C. h. Chang, J. Razink, M. M. Lerner, C. Fang, X. Ji, *Angew. Chem. Int. Ed.* **2020**, *132*, 20096.

[3] M. Wang, T. Li, Y. Yin, J. Yan, H. Zhang, X. Li, *Adv. Energy Mater.* **2022**, *12*, 2200728.

[4] W. Gao, S. Cheng, Y. Zhang, E. Xie, J. Fu, *Adv. Funct. Mater.* **2023**, *33*, 2211979.

[5] S. Bi, H. Wang, Y. Zhang, M. Yang, Q. Li, J. Tian, Z. Niu, *Angew. Chem. Int. Ed.* **2023**, *62*, e202312982.

[6] F. Chen, Z. Y. Leong, H. Y. Yang, *Energy Storage Mater.* **2017**, *7*, 189.

[7] S. Chai, J. Yao, Y. Wang, J. Zhu, J. Jiang, *Chem. Eng. J.* **2022**, *439*, 135676.

[8] X. Wu, Y. Qi, J. J. Hong, Z. Li, A. S. Hernandez, X. Ji, *Angew. Chem. Int. Ed.* **2017**, *56*, 13026.

[9] Q. Liu, F. Ye, K. Guan, Y. Yang, H. Dong, Y. Wu, Z. Tang, L. Hu, *Adv. Energy Mater.* **2023**, *13*, 2202908.

[10] X. Wen, J. Luo, K. Xiang, W. Zhou, C. Zhang, H. Chen, *Chem. Eng. J.* **2023**, *458*, 141381.

[11] Z. Bao, C. Lu, Q. Liu, F. Ye, W. Li, Y. Zhou, L. Pan, L. Duan, H. Tang, Y. Wu, *Nat. Commun.* **2024**, *15*, 1934.

[12] S. Dong, W. Shin, H. Jiang, X. Wu, Z. Li, J. Holoubek, W. F. Stickle, B. Key, C. Liu, J. Lu, *Chem* **2019**, *5*, 1537.

[13] H. Fei, F. Yang, Z. Jusys, S. Passerini, A. Varzi, *Adv. Funct. Mater.* **2024**, *34*, 2404560.

[14] S. Chen, Y. Ying, S. Wang, L. Ma, H. Huang, X. Wang, X. Jin, S. Bai, C. Zhi, *Angew. Chem. Int. Ed.* **2023**, *62*, e202301467.

[15] S. Chen, C. Peng, D. Zhu, C. Zhi, *Adv. Mater.* **2024**, *36*, 2409810.

[16] Q. Dong, M. Wei, Q. Zhang, L. Xiao, X. Cai, S. Zhang, Q. Gao, Y. Fang, F. Peng, S. Yang, *Chem. Eng. J.* **2023**, *459*, 141542.

[17] N. Tewari, S. B. Shivarudraiah, J. E. Halpert, *Nano Lett.* **2021**, *21*, 5578.

[18] T. Yang, H. Mao, Q. Zhang, C. Xu, Q. Gao, X. Cai, S. Zhang, Y. Fang, X. Zhou, F. Peng, *Angew. Chem. Int. Ed.* **2024**, *63*, e202403022.

[19] X. Zhang, W.-L. Song, M. Wang, J. Tu, H. Jiao, S. Jiao, *Energy Storage Mater.* **2022**, *45*, 586.

[20] Y.-H. Liu, J. Qu, W. Chang, C.-Y. Yang, H.-J. Liu, X.-Z. Zhai, Y. Kang, Y.-G. Guo, Z.-Z. Yu, *Energy Storage Mater.* **2022**, *50*, 334.

[21] Q. Zhang, M. Wei, Q. Dong, Q. Gao, X. Cai, S. Zhang, T. Yuan, F. Peng, Y. Fang, S. Yang, *J. Energy Chem.* **2023**, *79*, 83.
